# ARMC12 regulates spatiotemporal mitochondrial dynamics during spermiogenesis and is required for male fertility

**DOI:** 10.1073/pnas.2018355118

**Published:** 2021-02-03

**Authors:** Keisuke Shimada, Soojin Park, Haruhiko Miyata, Zhifeng Yu, Akane Morohoshi, Seiya Oura, Martin M. Matzuk, Masahito Ikawa

**Affiliations:** ^a^Research Institute for Microbial Diseases, Osaka University, 5650871 Osaka, Japan;; ^b^Graduate School of Medicine, Osaka University, 5650871 Osaka, Japan;; ^c^Center for Drug Discovery, Baylor College of Medicine, Houston, TX 77030;; ^d^Department of Pathology & Immunology, Baylor College of Medicine, Houston, TX 77030;; ^e^Graduate School of Pharmaceutical Sciences, Osaka University, 5650871 Osaka, Japan;; ^f^The Institute of Medical Science, The University of Tokyo, 1088639 Tokyo, Japan

**Keywords:** mitochondrial sheath formation, spermatogenesis, sperm mitochondrial dynamics, infertility

## Abstract

The mammalian sperm midpiece has a unique double-helical structure called the mitochondrial sheath that wraps tightly around the axoneme. Despite the remarkable organization of the mitochondrial sheath, the molecular mechanisms involved in mitochondrial sheath formation are unclear. In the process of screening testis-enriched genes for functions in mice, we identified armadillo repeat-containing 12 (ARMC12) as an essential protein for mitochondrial sheath formation. Here, we engineered *Armc12*-null mice, FLAG-tagged *Armc12* knock-in mice, and TBC1 domain family member 21 (*Tbc1d21*)-null mice to define the functions of ARMC12 in mitochondrial sheath formation in vivo. We discovered that absence of ARMC12 causes abnormal mitochondrial coiling along the flagellum, resulting in reduced sperm motility and male sterility. During spermiogenesis, sperm mitochondria in *Armc12*-null mice cannot elongate properly at the mitochondrial interlocking step which disrupts abnormal mitochondrial coiling. ARMC12 is a mitochondrial peripheral membrane protein and functions as an adherence factor between mitochondria in cultured cells. ARMC12 in testicular germ cells interacts with mitochondrial proteins MIC60, VDAC2, and VDAC3 as well as TBC1D21 and GK2, which are required for mitochondrial sheath formation. We also observed that TBC1D21 is essential for the interaction between ARMC12 and VDAC proteins in vivo. These results indicate that ARMC12 uses integral mitochondrial membrane proteins VDAC2 and VDAC3 as scaffolds to link mitochondria and works cooperatively with TBC1D21. Thus, our studies have revealed that ARMC12 regulates spatiotemporal mitochondrial dynamics to form the mitochondrial sheath through cooperative interactions with several proteins on the sperm mitochondrial surface.

## Significance

Although formation of the mitochondrial sheath is a critical process in the formation of mature spermatozoa, the molecular mechanisms involved in mitochondrial sheath genesis remain unclear. Using gene-manipulated mice, we discovered that ARMC12 regulates spatiotemporal “sperm mitochondrial dynamics” during mitochondrial sheath formation through interactions with mitochondrial proteins MIC60, VDAC2, and VDAC3 as well as testis-specific proteins TBC1D21 and GK2. In addition, we demonstrated that ARMC12-interacting proteins TBC1D21 and GK2 are also essential for mitochondrial sheath formation. Our paper sheds light on the molecular mechanisms of mitochondrial sheath formation and the regulation of sperm mitochondrial dynamics, allowing us to further understand the biology of spermatogenesis and the etiology of infertility in men.

Fertilization, the union of female gametes (oocytes) with their male counterpart (spermatozoa), is essential for the continuation of species. Sperm, or spermatozoa, consists of a compact, haploid head attached to a flagellum that provides the locomotive force required to deliver the haploid male genome to the egg. The flagellum is divided into a midpiece, principal piece, and end-piece region, and the mammalian sperm midpiece is characterized by the mitochondrial sheath that packs tightly around the axoneme and the outer dense fibers (1). Because this mitochondrial sheath structure shows remarkable regularity in its organization (2), it is unsurprising that disruption of this structure leads to reduced sperm motility and infertility in mice (3–5). Likewise, morphological abnormalities in mitochondrial sheath are sometimes observed in infertile men (6). These findings suggest that proper mitochondrial sheath formation is essential for fertility in both humans and mice.

The formation of the mitochondrial sheath in mammals occurs late in spermatogenesis (7). In this process, spherical-shaped mitochondria are recruited from the cytoplasm and line up around the flagellum. These spherical-shaped mitochondria elongate laterally to become crescent-like in shape. Subsequently, crescent-like mitochondria elongate continuously to coil tightly around the flagellum in a double helical structure (2, 8). Although formation of this well-organized mitochondrial sheath has been described ultrastructurally, the molecular mechanisms involved in mitochondrial sheath formation remain unclear due to a lack of good animal models and the identification of key pathway proteins.

Because the mitochondrial sheath structures in humans and mice have common characteristics (9) and ultrastructural microscopic analysis of mitochondrial sheath formation has been established (8), mice are an excellent animal model for the study of mitochondrial sheath formation. To unveil the factor(s) involved in mitochondrial sheath formation, creating and analyzing male mice carrying null mutations is a feasible approach because we can observe protein function directly, and as yet no culture systems exist that produce fully functional spermatozoa in vitro. Therefore, we generated knockout (KO) male mice using CRISPR/Cas9-based genome engineering and screened their phenotypes (10–12) to find factors involved in mitochondrial sheath formation. In addition, we analyzed the functions of identified proteins to unveil the molecular mechanisms of mitochondrial sheath formation.

In the process of conducting functional screens of testis-enriched genes in mice, we identified armadillo repeat-containing 12 (ARMC12) as an essential protein for mitochondrial sheath formation. ARMC12 belongs to the ARM family which consists of proteins with similar ARM-repeat motifs of ∼40 amino acids that form three α-helices (13). Several ARM family proteins were reported to be related to mitochondria (14–16) or spermatogenesis (17, 18), but there have been no reports related to mitochondrial sheath formation. ARMC12 was reported to be highly expressed in clinical neuroblastoma specimens and to drive the growth and aggressiveness of neuroblastoma cell lines (19). However, because *Armc12* shows testis-enriched expression according to the Mouse ENCODE Project (20), the pathophysiological role of ARMC12 in this cancer is likely due to its ectopic expression.

Here, we engineered the *Armc12* locus to produce both KO mice and FLAG-tagged knock-in (KI) mice to define the functions of ARMC12 in mitochondrial sheath formation in vivo. Using these lines, we revealed that ARMC12 is essential for male fertility and proper mitochondrial elongation to coil along the flagellum during spermiogenesis. Our study reveals the molecular mechanisms involved in mitochondrial sheath formation during spermiogenesis and uncovers an aspect of “sperm mitochondrial dynamics” that is essential for generating functional spermatozoa and fertility.

## Results

### ARMC12 Is an Evolutionarily Conserved Testis-Enriched Protein.

The Ensembl database (http://asia.ensembl.org/index.html) shows that ARMC12 is highly conserved among reptiles, marsupials, and mammals. Representative amino acid sequences of ARMC12 from reptiles (turtle), marsupials (opossum), and mammals (dog, mouse, and human) contain the conserved ARM domains (*SI Appendix*, Fig. S1*A*) which are tandem copies of a degenerate protein sequence motif that forms a conserved three-dimensional structure (21).

**Fig. 1.**
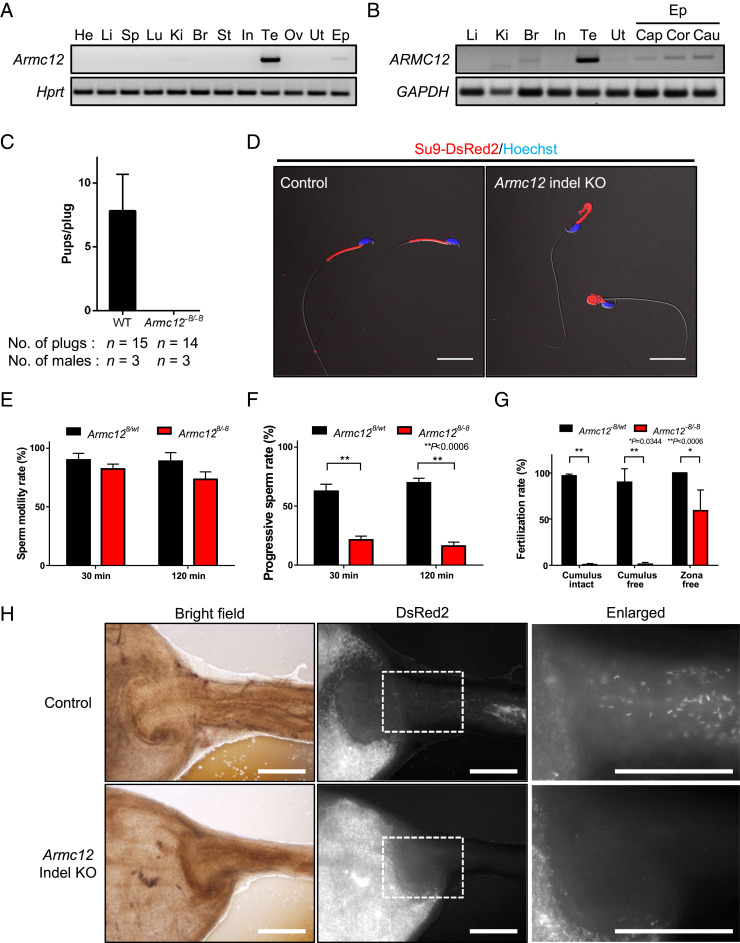
*Armc12* indel KO male mice are sterile due to reduced sperm motility and abnormal sperm morphology. (*A* and *B*) RT-PCR for *Armc12* from various mouse tissues (*A*) and RT-PCR for *ARMC12* from various human tissues (*B*) (He, heart; Li, liver; Sp, spleen; Lu, lung; Ki, kidney; Br, brain; St, stomach; In, intestine; Te, testis; Ov, ovary; Ut, uterus; Ep, epididymis; Cap, caput; Cor, corpus; Cau, Cauda). *Hprt* and *GAPDH* were used as controls. (*C*) Pregnancy rate of control and *Armc12* indel KO male mice. (*D*) Sperm morphology of control and *Armc12* indel KO mice with RBGS transgenes, which express mitochondria-targeted DsRed2 (red). Nuclei were stained with Hoechst 33342 (blue). (Scale bars, 20 µm.) (*E*) Sperm motility from control and *Armc12* indel KO mice. Error bars represent SD (*n* = 3). (*F*) Progressive sperm rate of sperm from control and *Armc12* indel KO mice. **P < 0.01, Student’s *t* test; error bars represent SD (*n* = 3). (*G*) Fertilization rate of IVF using control and *Armc12* indel KO spermatozoa. Three types of oocytes (cumulus-intact, cumulus-free, and zona-free) were used for IVF. *P < 0.05, **P < 0.01, Student’s *t* test; error bars represent SD (*n* = 3). (*H*) Imaging of spermatozoa inside the female reproductive tract 2 to 3 h after observing vaginal plugs. (*Left*) Reproductive organs under normal bright-field conditions. (*Middle*) Red fluorescence of RBGS spermatozoa in the female reproductive tract. (*Right*) Magnified images of the boxed areas. Fluorescent images were false-colored using ImageJ software. (Scale bars, 500 µm.)

To determine the expression profile of *Armc12*, we performed RT-PCR using multiple tissues from adult mice. RT-PCR revealed that *Armc12* is expressed in testis and weakly in kidney and epididymis, but not in other tissues (Fig. 1*A*). To determine the temporal expression of *Armc12* in testis, we performed RT-PCR from early postnatal day testis to capture the leading edge of the first wave of spermatogenesis. *Armc12* begins to express around postnatal day 20 (*SI Appendix*, Fig. S1*B*), which roughly corresponds to the haploid, round spermatid stage of spermatogenesis. To determine possible functional conservation in humans, we performed RT-PCR using multiple tissues from humans and discovered that ARMC12 is expressed in testis and weakly in brain and epididymis (Fig. 1*B*). These results indicate that ARMC12 is a testis-enriched protein with predicted cross-species spatiotemporal and functional conservation during spermatogenesis.

### *Armc12* Knockout Mice Are Sterile.

To examine the functions of *Armc12* in vivo, we generated *Armc12* KO mice with the CRISPR/Cas9 system using a guide RNA which targets downstream of the start codon (*SI Appendix*, Fig. S2*A*). A mutant mouse line that had an 8-bp deletion was used for this study (*SI Appendix*, Fig. S2*B*). *Armc12*^*−8/−8*^ mice are viable and show no overt abnormalities. To test the fertility of the mice, individual males (WT and homozygous) were housed with WT females for 2 mo. Although vaginal plugs were observed 14 times, no pups were born from *Armc12*^*−8/−8*^ male mice (Fig. 1*C*). In contrast, *Armc12*^*−8/−8*^ female mice are fertile with the average litter size being 7.4 ± 1.2 (mean ± SD, *n* = 13). Thus, ARMC12 is required for male fertility.

### *Armc12* Null Spermatozoa Cannot Pass through the Uterotubal Junction Due to Abnormal Sperm Morphology and Motility.

To reveal the cause(s) of the infertility of the *Armc12* KO male mice, we initially examined spermatozoa obtained from *Armc12* KO male mice. In contrast to normal-appearing spermatozoa from control (heterozygous mutant or WT) mice, *Armc12* KO spermatozoa collected from the cauda epididymis had abnormal bending of the tail and a disorganized mitochondrial sheath (Fig. 1*D* and *SI Appendix*, Fig. S2*C*). Morphological and histological analysis of the testes of KO mice revealed no significant differences in gross appearance, testis weight, and testis histology (*SI Appendix*, Fig. S2 *D*–*F*). Because of the bending of the tail and mitochondrial defects in null spermatozoa, we examined sperm motility using computer-assisted spermatozoa analysis. Although the percentage of motile sperm in *Armc12* KO mice was similar to controls (Fig. 1*E*), the progressive sperm rate dramatically decreased in *Armc12* KO spermatozoa (Fig. 1*F* and Movies S1 and S2). In addition, *Armc12* KO spermatozoa showed significantly lower values in average path velocity (VAP), straight line velocity (VSL), and curvilinear velocity (VCL) at both 30 and 120 min of incubation (*SI Appendix*, Fig. S3*A*). Thus, the abnormal sperm morphology in the KO spermatozoa appeared to cause defects in sperm motility.

To analyze fertilizing ability of *Armc12* KO spermatozoa, we performed in vitro fertilization (IVF) and found that the fertilization rate of *Armc12* KO spermatozoa with both cumulus-intact and cumulus-free oocytes was essentially absent (Fig. 1*G*). However, 63% of the zona pellucida (ZP)-free oocytes (43/68) were fertilized with *Armc12* KO spermatozoa (Fig. 1*G*). To confirm the ability of KO spermatozoa to bind to the ZP, we incubated cumulus-free oocytes with spermatozoa in vitro (22) and discovered that *Armc12* KO spermatozoa rarely bind to the ZP (*SI Appendix*, Fig. S3 *B* and *C*). To affirm the ability of KO spermatozoa to undergo the acrosome reaction, we analyzed IZUMO1 localization 2 h after incubation in capacitation medium. We observed that both control and KO spermatozoa similarly underwent the acrosome reaction (*SI Appendix*, Fig. S3*D*). These results indicate that *Armc12* KO spermatozoa can undergo the acrosome reaction and fuse with oocytes, but they have a defect in sperm-ZP binding.

To study the infertility phenotype in more detail, we examined sperm migration into the oviduct using *Armc12* KO male mice with red body green sperm (RBGS) transgenes that express enhanced green fluorescent protein in the acrosome and DsRed2 in the mitochondria (23). WT spermatozoa can transit into the oviduct through the uterotubal junction (UTJ); however, while KO sperm can be observed in the uterus near the UTJ, no *Armc12* KO spermatozoa pass through the UTJ into the oviduct (Fig. 1*H*). Because the presence and processing of the sperm head protein ADAM3 is essential for passing through the UTJ (24–26), we examined ADAM3 by Western blot (WB) analysis and found that ADAM3 processing is normal in *Armc12* KO mice (*SI Appendix*, Fig. S3*E*). These results indicate that *Armc12* KO male mice are sterile due to an impairment of sperm passage through the UTJ despite proper ADAM3 processing. Because *Armc12* KO spermatozoa cannot maintain their head at the apex while swimming (Movie S2), the *Armc12*-null sperm head rarely has the chance to interact with the UTJ epithelium or bind to the ZP.

### ARMC12 Is Essential for Proper Mitochondrial Elongation and Coiling along the Flagellum during Formation of the Mitochondrial Sheath.

**Fig. 2.**
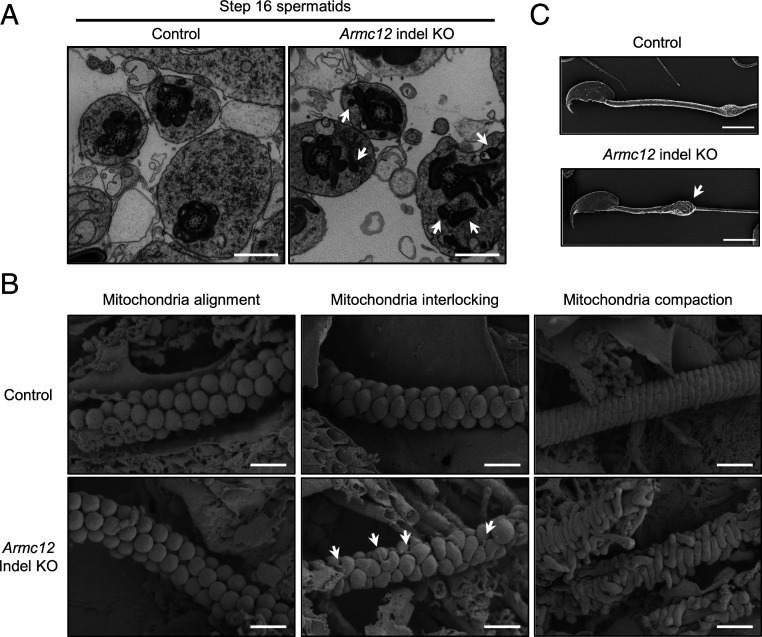
ARMC12 is essential for proper mitochondrial elongation and subsequent coiling along the flagellum during spermiogenesis. (*A*) Ultrastructural images of step 16 spermatids analyzed by TEM. Arrows indicate aberrant location of sperm mitochondria. (Scale bars, 1.0 μm.) (*B*) Development of mitochondrial sheath during spermiogenesis observed by SEM. Arrows indicate exposed mitochondrial tips. (Scale bars, 1.0 μm.) (*C*) Sperm morphology of control and *Armc12* indel KO spermatozoa collected from the cauda epididymis. Samples were observed by SEM. Arrow indicates abnormal uncoiled mitochondria that shed from the midpiece. (Scale bars, 5.0 µm.)

To analyze the emergence of morphological defects in *Armc12*-null spermatozoa, spermatozoa were collected from the three sections of the epididymis (caput, corpus, and cauda). We discovered that the majority of *Armc12* KO spermatozoa were already morphologically abnormal in the caput epididymis and increased to 100% during sperm transit through the corpus and cauda epididymis (*SI Appendix*, Fig. S4*A*). Then, we performed ultrastructural analysis of control and mutant spermatids using both transmission electron microscopy (TEM) and scanning electron microscopy (SEM) to analyze the defects in *Armc12* KO spermatozoa in more detail. TEM observation revealed that spermatozoa from *Armc12*^*−8/−8*^ males show mitochondrial disorganization in step 16 spermatids (Fig. 2*A*). We then performed a freeze-fracture method with SEM to observe mitochondrial sheath development (8). Although we observed proper alignment of spherical-shaped mitochondria in *Armc12* KO spermatids (Fig. 2*B*, *Lower Left*), aberrant mitochondrial arrangement was observed at the next step (Fig. 2*B*, *Lower Middle*, and *SI Appendix*, Fig. S4*B*). During these steps, each spherical-shaped mitochondrion in control spermatids elongates laterally and becomes crescent-like in shape (Fig. 2*B*, *Upper Middle*) to form a well-arranged structure as each sperm mitochondrion is interlocked by multiple adjacent mitochondria. Due to the aberrant mitochondrial interlocking structure, we could find the mitochondrial “tips” in *Armc12* KO spermatids that are not observed in WT. After the mitochondrial-interlocking step, sperm mitochondria continue to elongate laterally to form the mitochondrial sheath (Fig. 2*B*, *Upper Right*). However, although the sperm mitochondria in the *Armc12* KO spermatids can elongate near the flagellum in this step, they cannot coil along the flagellum (Fig. 2*B*, *Lower right*, and *SI Appendix*, Fig. S4*B*). These findings indicate that ARMC12 is essential for proper mitochondrial elongation to coil along the flagellum at the interlocking step, suggesting that ARMC12 regulates spatiotemporal mitochondrial dynamics during mitochondrial sheath formation. To observe mitochondrial behavior after sperm maturation, SEM analysis was conducted using spermatozoa from the cauda epididymis. *Armc12* KO mature spermatozoa have abnormal uncoiled mitochondria (Fig. 2*C*) due to shedding of mitochondria which could not coil along the flagellum. Thus, the morphological abnormalities observed in *Armc12* KO spermatozoa are due to an abnormal mitochondrial sheath formation during spermiogenesis.

To check mitochondrial function in the absence of ARMC12, we evaluated sperm mitochondrial activity of *Armc12* KO by the fluorescent carbocyanine dye JC-1, which labels mitochondria with high membrane potential “orange” and mitochondria with low membrane potential “green” (27). Control and *Armc12* KO spermatozoa demonstrate bright orange fluorescence (JC-1 multimer) at the midpiece (*SI Appendix*, Fig. S4*C*), indicating high membrane potential of the inner mitochondrial membrane. Quantitative fluorescence image analysis reveals a statistically equal orange/green fluorescence ratio between control and KO spermatozoa (1.9 ± 0.5 vs. 1.8 ± 0.4, respectively). These results indicate that the morphological defects in the *Armc12* KO spermatozoa have no effect on mitochondrial activity.

### *Armc12* Large Deletion KO Mice Phenocopy *Armc12* Indel KO Mice.

Since an alternative splicing variant or a truncated protein might be expressed in the indel KO mice, we could not conclude that *Armc12*^*−8/−8*^ indel KO mice have completely lost the ARMC12 protein. To assess the expression level of ARMC12 protein in *Armc12*^*−8/−8*^ KO mice, we evaluated several ARMC12 antibodies, but we could not obtain a good antibody for analyzing ARMC12 protein. To reaffirm that this phenotype was solely a result of the lack of ARMC12 protein, we engineered mutant mice that carry a large deletion (LD) of *Armc12*. A mutant line that had an 8,015-bp deletion lacking ARMC12 amino acids 11 to 340 was generated using two guide RNAs (*SI Appendix*, Fig. S5 *A* and *B*). *Armc12*^*LD/LD*^ mice were viable and showed no overt abnormalities. We performed RT-PCR of full-length *Armc12* transcript using both indel and LD KO testis RNA and found that *Armc12* LD KO mice lose *Armc12* expression completely (*SI Appendix*, Fig. S5*C*), indicating that *Armc12* LD KO mice fail to synthesize ARMC12. Using this *Armc12* LD KO male mice, we evaluated their phenotype. *Armc12* LD KO male mice are sterile (*SI Appendix*, Fig. S5*D*). *Armc12* LD KO spermatozoa collected from the cauda epididymis displayed both abnormal bending of the tail and a disorganized mitochondrial sheath, phenocopying the *Armc12* indel KO spermatozoa (*SI Appendix*, Fig. S5*E* and Movies S3 and S4). We also observed mitochondrial behavior of *Armc12* LD KO spermatids during spermiogenesis and found that it was indistinguishable from that of *Armc12* indel KO spermatids (*SI Appendix*, Fig. S5*F*). Thus, the phenotypes of *Armc12*^*−8/−8*^ and *Armc12*^*LD/LD*^ are identical, indicating that ARMC12 protein is functionally absent in both KO lines.

### ARMC12 Localizes to the Mitochondrial Outer Membrane of Spermatids.

**Fig. 3.**
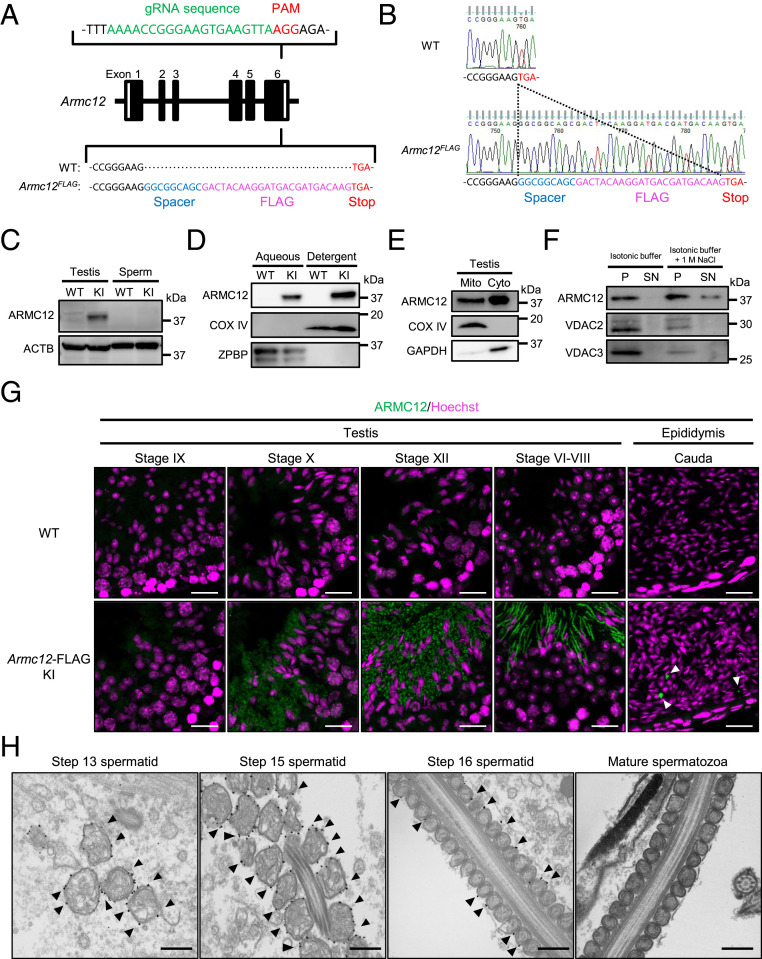
ARMC12 localizes to the mitochondrial outer membrane of spermatids. (*A*) Schematic diagram of FLAG-tagged alleles of endogenous *Armc12*. KI allele has a three-amino-acid spacer before the FLAG tag. (*B*) Control and *Armc12*^*FLAG*^ alleles. (*C*) WB analysis using lysates prepared from the testis and mature spermatozoa from WT and FLAG-tagged *Armc12* KI mice. ACTB was used as a loading control. (*D*) WB analysis using aqueous and detergent fractions of Triton X-114 extracts of the testicular cells. COX IV and ZPBP were used as membrane protein and cytosolic protein markers, respectively. (*E*) WB analysis using isolated mitochondrial and cytosolic fractions from FLAG-tagged *Armc12* KI testicular cells. COX IV and GAPDH were used as mitochondrial protein and cytosolic protein markers, respectively. (*F*) Mitochondria purified from *Armc12*-FLAG KI testicular cells were incubated with isotonic buffer or high salt (1 M NaCl) followed by centrifugation to isolate pellet (P) and supernatant (SN) fractions. Equal volumes of protein from each fraction were subjected to WB analysis. VDAC2 and VDAC3 were used as control of integral mitochondrial membrane protein. (*G*) Immunostaining of FLAG-tagged ARMC12 in *Armc12*-FLAG KI mouse testis and epididymis. Arrowheads indicate ARMC12 that are not localized in the sperm midpiece. Fluorescent images were false colored using ImageJ software. Spermatogenic stages were identified by the nuclear morphology. (Scale bars, 20 µm.) (*H*) Detection of immunolabeled FLAG-tagged ARMC12 in testis by TEM using anti-FLAG antibody incubated with 1.4-nm gold-particle–conjugated secondary antibody (arrowheads). (Scale bars, 500 nm.)

To define the spatiotemporal and subcellular localization of ARMC12, we produced FLAG-tagged *Armc12* KI mice by the CRISPR/Cas9 system using embryonic stem cells as previously described (28). The targeting vector for the KI allele was designed as shown (Fig. 3*A*). FLAG-tagged KI mice were obtained from chimeric male mice, and subsequent mating resulted in homozygous FLAG-tagged mutant mice (*Armc12*^*FLAG/FLAG*^, Fig. 3*B*). We confirmed that *Armc12*^*FLAG/FLAG*^ mice have no fertility problems in both males and females. When we performed WB analysis, FLAG-tagged ARMC12 was detected in extracts from testis, but not from mature spermatozoa (Fig. 3*C*). To further localize ARMC12, testicular proteins isolated from KI mice were subjected to Triton X-114 fractionation to separate proteins into aqueous-phase and detergent-enriched proteins (24). After the fractionation, FLAG-tagged ARMC12 was observed in both phases (Fig. 3*D*). Because ARMC12 was observed in both phases and related to mitochondrial sheath formation, testicular cells from FLAG-tagged ARMC12 were separated into mitochondrial and cytosolic fractions. WB analysis revealed that ARMC12 was detected in both fractions (Fig. 3*E*). Then, we tested if ARMC12 could be extracted from mitochondria using high-salt buffer (1.0 M NaCl in isotonic buffer). ARMC12 protein was removed from the membranes and recovered in the supernatant (Fig. 3*F*), indicating that ARMC12 is a mitochondrial peripheral membrane protein.

We next determined the spatiotemporal distribution of ARMC12 by immunostaining. ARMC12 is expressed in steps 10 to 16 spermatids and localizes in the midpiece of step 16 spermatids (Fig. 3*G*). However, ARMC12 was not observed in the midpiece of spermatozoa in cauda epididymis. Instead, the ARMC12 is located outside spermatozoa (Fig. 3*G*, arrowheads). This implies that ARMC12 is discarded during epididymal migration. At the ultrastructural level, immunolabeled ARMC12 localizes to the mitochondrial outer membrane in spermatids (Fig. 3*H*, arrowheads). However, ARMC12 was not detected in mature spermatozoa from the cauda epididymis (Fig. 3*H*, rightmost), consistent with the WB analysis (Fig. 3*C*) and immunostaining (Fig. 3*G*). Thus, ARMC12 localizes to the mitochondrial outer membrane and functions in mitochondrial sheath development during spermiogenesis but disappears in mature spermatozoa after the mitochondrial sheath is organized.

### The N Terminus of ARMC12 Functions in Mitochondrial Adherence In Vitro.

**Fig. 4.**
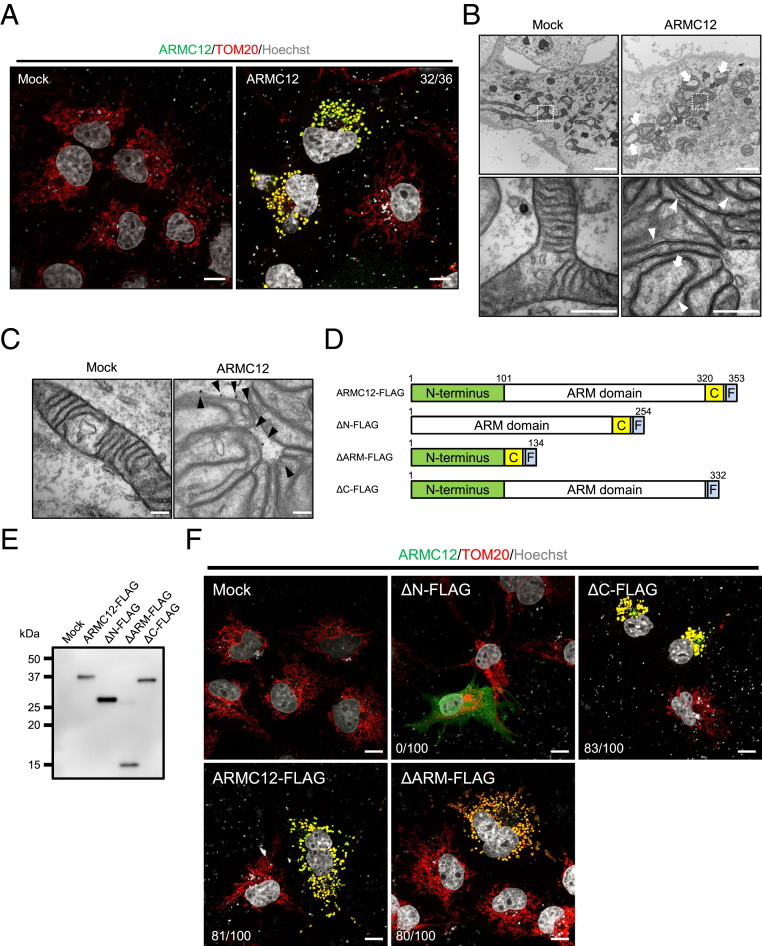
ARMC12 functions as an adherence factor between mitochondria in cultured cells. (*A*) COS-7 cells transiently expressing FLAG-tagged ARMC12 protein were stained with FLAG (green) and TOM20 (red) to visualize ARMC12 and mitochondria, respectively. Hoechst 33342 (white) was used to visualize the nuclei. (Scale bars, 10 µm.) The numbers in the upper right corner indicate the number of cells with mitochondrial aggregation out of the number of cells with FLAG fluorescence. (*B*) TEM micrographs of COS-7 cells overexpressing a control vector (*Left*) or ARMC12 protein (*Right*). (*Lower*) Magnified images of the boxed areas. Arrows indicate mitochondria with reduced cristae. Arrowheads indicate the border between mitochondria. (Scale bars, 2.0 μm and 500 nm, respectively.) (*C*) Immunolabeled detection of transiently expressing FLAG-tagged ARMC12 in COS-7 cells was observed by TEM using anti-FLAG antibody combined with 1.4 nm gold-particle–conjugated secondary antibody (arrowheads). (Scale bars, 200 nm.) (*D*) Schematic of various truncated ARMC12 vectors. FLAG-tag was linked posterior to the C-terminal of ARMC12. Green and yellow boxes show the N terminus and C terminus of ARMC12, respectively. Light blue boxes indicate FLAG tag. A five-amino-acid spacer is inserted between the C terminus and FLAG tag. (*E*) HEK293T cells were transfected with plasmids encoding FLAG-tagged full length ARMC12 and truncated forms of ARMC12. Cell lysates were analyzed by immunoblotting using an anti-FLAG antibody. (*F*) COS-7 cells transiently expressing truncated ARMC12 were stained with FLAG (green) and TOM20 (red) to visualize ARMC12 and mitochondria, respectively. Hoechst 33342 (white) was used to visualize the nuclei. (Scale bars, 10 µm.) The numbers in the lower left corner indicate the number of cells with mitochondrial aggregation out of the number of cells with FLAG fluorescence.

To further define the functions of ARMC12, we transiently expressed FLAG-tagged ARMC12 protein in COS-7 cells and examined mitochondrial morphology. We found that ARMC12 colocalizes with the mitochondrial marker TOM20 and induces morphological changes of the mitochondria (Fig. 4*A*). Furthermore, we observed aggregation of mitochondria in COS-7 cells after *Armc12* overexpression using time-lapse video microscopy (Movie S5). Using TEM, we observed mitochondrial clusters in ARMC12-expressing COS-7 cells (Fig. 4*B*). Because multiple mitochondria adhere tightly, there were no gaps between mitochondria in ARMC12-expressing cells (Fig. 4*B*, arrowheads). This result implies that ARMC12 acts as an adherence factor between mitochondria. Furthermore, mitochondria in clusters show markedly reduced cristae (Fig. 4*B*, arrows). We also performed immunolabeling of FLAG-tagged ARMC12 in COS-7 cells and analyzed its localization using TEM. As expected, ARMC12 localized to the mitochondrial outer membrane in COS-7 cells (Fig. 4*C*, arrowheads).

To determine the ARMC12 domain functions, various FLAG-tagged *Armc12* deletion mutants were constructed (Fig. 4*D*). Transfection of the WT and truncated ARMC12 constructs and immunoblot analysis using anti-FLAG antibody confirmed that the truncated protein sizes were proportional to their coding sequence (Fig. 4*E*). Immunostaining of the transfected cells revealed that truncated ARMC12 without either the ARM domain (ΔARM-FLAG) or the C terminus (ΔC-FLAG) continues to induce formation of mitochondrial clusters, whereas ARMC12 lacking the N terminus (Δ*N*-FLAG) does not induce aggregation (Fig. 4*F*). Thus, the N terminus of ARMC12 is indispensable for ARMC12 function involved in mitochondrial clustering, even though the N terminus of ARMC12 has no domain information.

### ARMC12 Interacts with TBC1D15 to Target Mitochondria in Cultured Cells.

To uncover the molecular mechanisms of ARMC12 function in mitochondrial clustering, we searched for ARMC12-interacting proteins using a second-generation proximity-dependent biotin identification system (BioID2) (29, 30). A biotin ligase (BirA)-fused ARMC12 (ARMC12-BioID2) construct was produced as shown (*SI Appendix*, Fig. S6*A*), and the ARMC12-BioID2-expressing plasmid was transfected into HEK293T cells in the presence of excess biotin. BirA-fused ARMC12 induces mitochondrial clusters (*SI Appendix*, Fig. S6*B*), indicating that the functions of BirA-fused ARMC12 are similar to those of FLAG-tagged ARMC12. After transfection of the ARMC12-BioID2 vector, cell lysates were blotted using a horseradish-peroxidase–conjugated streptavidin or anti-FLAG antibody (*SI Appendix*, Fig. S6 *C* and *D*). Several specific bands except for BirA-fused ARMC12 were observed after ARMC12-BioID2 transfection (*SI Appendix*, Fig. S6*C*), suggesting that multiple proteins were biotinized by BirA-fused ARMC12. We also confirmed that biotinized proteins colocalized with ARMC12 (*SI Appendix*, Fig. S6*E*). Cell lysates from BirA-fused ARMC12 transfected cells were subjected to pull down by streptavidin, and mass spectrometry (MS) analysis of these proteins was performed. Candidate ARMC12-interacting proteins included several proteins reported to be in mitochondria or interact with mitochondria (*SI Appendix*, Fig. S6*F*).

We selected TBC1D15, DNM1L, OCIAD1, and AKAP1 to check their direct interactions with ARMC12. Cell lysates obtained from HEK293T cells transfected with FLAG-tagged ARMC12 were subjected to immunoprecipitation using an anti-FLAG antibody and then analyzed by immunoblotting using anti-TBC1D15, DNM1L, OCIAD1, and AKAP1 antibodies. ARMC12 interacts with endogenous TBC1D15, but not DNM1L, OCIAD1, and AKAP1 (*SI Appendix*, Fig. S6*G*). TBC1D15 is known to interact with the mitochondrial transmembrane protein mitochondrial fission 1 (FIS1) (31, 32), suggesting that ARMC12 is anchored on the mitochondrial outer membrane via TBC1D15 and FIS1. However, a direct interaction between ARMC12 and FIS1 could not be observed in transfected cells (*SI Appendix*, Fig. S7*A*). Next, we confirmed the interactions between various truncated forms of ARMC12 and TBC1D15, revealing that TBC1D15 interacts with both ΔARM-FLAG and ΔC-FLAG, but not with Δ*N*-FLAG (*SI Appendix*, Fig. S7*B*), which is coincident with the results of immunostaining analysis (Fig. 4*F*). Thus, the N terminus of ARMC12 is essential not only for mitochondrial clustering, but also for binding to TBC1D15 to target mitochondria.

### ARMC12 Interacts with Several Proteins to Form the Mitochondrial Sheath.

**Fig. 5.**
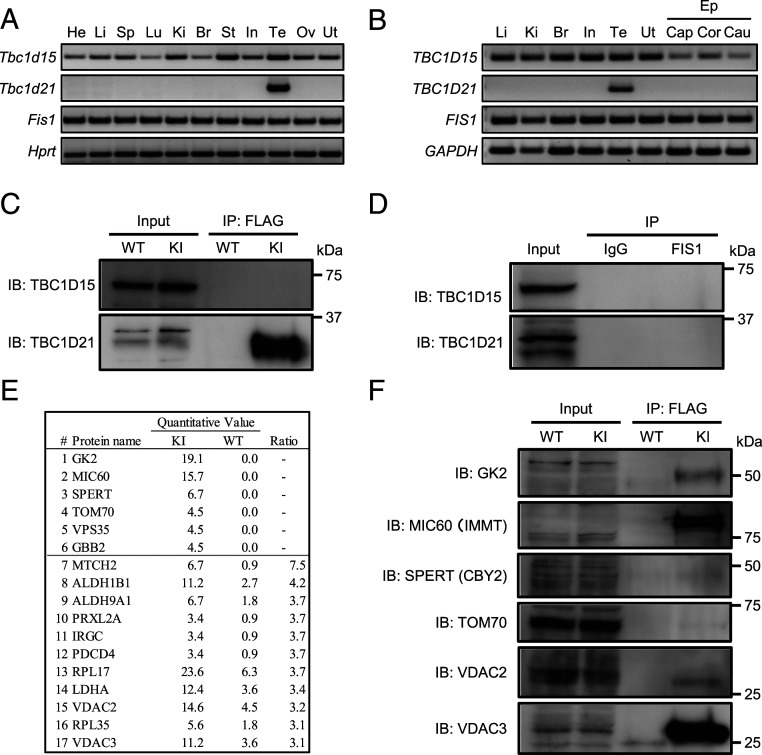
ARMC12 interacts with several proteins to form the mitochondrial sheath during spermiogenesis. (*A* and *B*) RT-PCR for *Tbc1D15*, *Tbc1d21*, and *Fis1* from various mouse tissues (*A*) and RT-PCR for *TBC1D15*, *TBC1D21*, and *FIS1* from various human tissues (*B*) (He, heart; Li, liver; Sp, spleen; Lu, lung; *K*i, kidney; Br, brain; St, stomach; In, intestine; Te, testis; Ov, ovary; Ut, uterus; Ep, epididymis; Cap, caput; Cor, corpus; Cau, Cauda). *Hprt* and *GAPDH* were used as controls. (*C* and *D*) The co-IP followed by Western blot analysis was performed using lysates collected from WT or FLAG-tagged *Armc12* KI mouse TGC. Immunoprecipitated proteins by anti-FLAG antibody (*C*) or anti-FIS1 antibody (*D*) were analyzed by WB using anti-TBC1D15 and TBC1D21 antibodies. IgG was used as a negative control for the IP. (*E*) List of identified proteins by MS analysis in vivo. Mitochondria were isolated from WT or FLAG-tagged *Armc12* KI testis, and proteins were extracted from the isolated mitochondria. The proteins were immunoprecipitated using anti-FLAG antibody and identified by MS analysis. This list shows proteins either identified only in FLAG-tagged *Armc12* KI mice (quantitative value >3.0) or highly identified in KI mice (ratio >3.0 as compared with WT) are listed. (*F*) Co-IP followed by WB analysis were performed using lysates collected from WT or FLAG-tagged *Armc12* KI mice TGC. Immunoprecipitated proteins by anti-FLAG antibody were analyzed by WB using anti-GK2, MIC60, SPERT, TOM70, VDAC2, and VDAC3 antibodies.

To define the molecular mechanisms of ARMC12 function in vivo, we analyzed ARMC12-interacting proteins using lysates collected from testicular germ cells (TGC). Before checking the interaction, we analyzed the expression patterns of *Tbc1d15* and *Fis1* in mice and humans and found that they were ubiquitously expressed as expected (Fig. 5 *A* and *B*). Then, we checked the interaction between ARMC12 and TBC1D15 by coimmunoprecipitation (co-IP) using TGC. The co-IP and subsequent WB analysis revealed that ARMC12 does not interact with TBC1D15 in TGC (Fig. 5*C*). Because TBC1 domain family protein TBC1D21 is the only TBC1 domain family protein showing testis-enriched expression in mice (20) and disruption of *Tbc1d21* causes male infertility with morphological abnormalities of the sperm mitochondria (33), we focused on TBC1D21. We performed RT-PCR and confirmed that *Tbc1d21* shows testis-enriched expression in mice and humans (Fig. 5 *A* and *B*). Subsequently, co-IP analysis revealed that ARMC12 interacts with TBC1D21 in mouse TGC (Fig. 5*C*). Because TBC1D15 interacts with mitochondrial outer membrane protein FIS1 and regulates mitochondrial morphology (31), we analyzed the interaction between TBC1D15, TBC1D21, and FIS1. However, interaction between TBC1D15, TBC1D21, and FIS1 could not be observed in mouse TGC (Fig. 5*D* and *SI Appendix*, Fig. S8*A*), suggesting that ARMC12 acts differently in vivo compared to in vitro.

To determine the ARMC12-interacting proteins which are required for targeting sperm mitochondria, mitochondria were isolated from FLAG-tagged *Armc12* KI mouse testes, and proteins were extracted and immunoprecipitated using anti-FLAG antibody to perform MS analysis. The MS analysis revealed that several mitochondria-related proteins were highly detected in the immunoprecipitates (Fig. 5*E*). From the detected proteins, we chose GK2, MIC60 (IMMT, mitofilin), SPERT (CBY, nurit), TOM70 (TOMM70), VDAC2, and VDAC3 to further characterize their interactions with ARMC12. We first confirmed that *Gk2* and *Cby2* show testis-enriched expression in both mice and humans, while *Immt*, *Tomm70*, *Vdac2*, and *Vdac3* show ubiquitous expression including testis (*SI Appendix*, Fig. S8 *B* and *C*), indicating that all proteins exist in the testis. We then found that ARMC12 interacts with GK2, MIC60, VDAC2, and VDAC3 in TGC, but not with SPERT and TOM70 (Fig. 5*F*). Thus, ARMC12 interacts with several proteins in the testis to regulate sperm mitochondrial dynamics during mitochondrial sheath formation.

### Disruption of ARMC12-Interacting Protein TBC1D21 Causes Disorganization of Mitochondrial Sheath Formation.

**Fig. 6.**
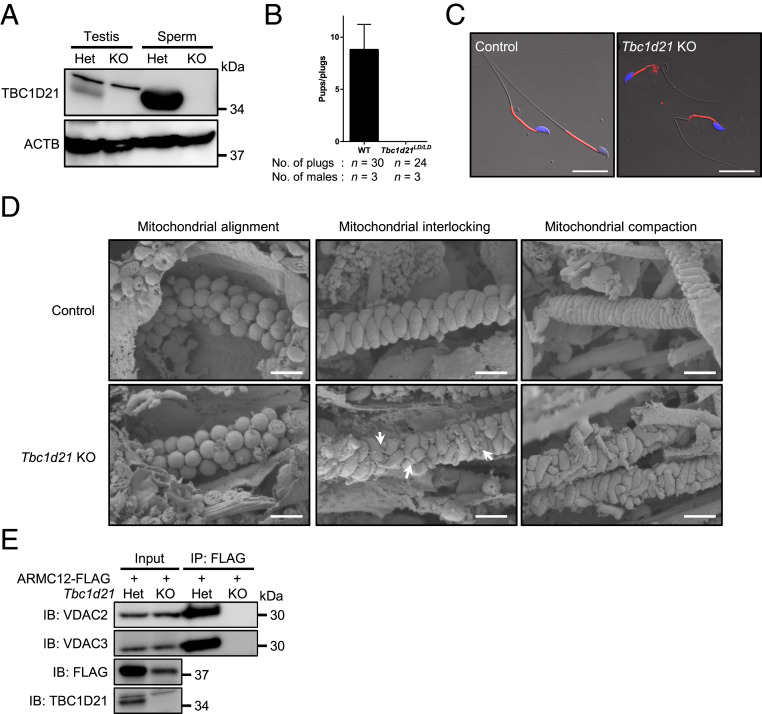
Disruption of TBC1D21 causes disorganization of mitochondrial sheath formation. (*A*) WB analysis of TBC1D21 using proteins obtained from control and *Tbc1d21* KO mouse. ACTB was used as a loading control. (*B*) Pregnancy rate of control and *Tbc1d21* KO male mice. (*C*) Sperm morphology of control and *Tbc1d21* KO mice with RBGS transgenes, which express mitochondria-targeted DsRed2 (red). Nuclei were stained with Hoechst 33342 (blue). (Scale bars, 20 µm.) (*D*) Mitochondrial sheath development during spermiogenesis observed by SEM. Arrows indicate exposed mitochondrial tips. (Scale bars, 1.0 µm.) (*E*) The co-IP followed by WB analysis was performed using lysates collected from *Tbc1d21* heterozygous or homozygous KO mouse TGC with a FLAG-tagged *Armc12* KI allele. Immunoprecipitated proteins by anti-FLAG antibody were analyzed by WB using anti-VDAC2 and VDAC3 antibodies. WB analysis using anti-FLAG and TBC1D21 was performed to confirm the existence of FLAG-tagged KI allele and disappearance of TBC1D21.

ARMC12-interacting protein genes *GK2* and *TBC1D21* shows testis-enriched expression in both mice and humans (Fig. 5 *A* and *B* and *SI Appendix*, Fig. S8 *B* and *C*), suggesting that these proteins have important roles in mitochondrial sheath formation. Previous study showed that knockout of *Gk2* induced disorganization of the mitochondrial sheath formation and that GK2 is essential for proper arrangement of mitochondria at the mitochondrial interlocking step (3). Based on our ARMC12-TBC1D21 interaction finding and the testis-restricted expression of *Tbc1d21*, we generated genome-edited mice lacking *Tbc1d21*. A mutant line that had an 11,311-bp deletion was generated using two guide RNAs (*SI Appendix*, Fig. S8 *D* and *E*). *Tbc1d21*^*LD/LD*^ mice are viable and show no overt abnormalities. Using WB analysis, we found that TBC1D21 is expressed in both testis and spermatozoa in control mice but depleted in *Tbc1d21* KO male testis and spermatozoa (Fig. 6*A*). Next, *Tbc1d21* KO male mice were subjected to a mating test, and we found that *Tbc1d21* KO male mice are sterile (Fig. 6*B*) and that KO spermatozoa from the cauda epididymis have an abnormality in the midpiece (Fig. 6*C*), similar to a recently published study (33). To check mitochondrial sheath formation in *Tbc1d21* KO male mice, we performed freeze-fracture SEM analysis. Although there were no problems in the mitochondrial alignment step (Fig. 6*D*, *Lower Left*), *Tbc1d21* KO spermatids show an abnormal interlocking structure (Fig. 6*D*, *Lower Middle*). In addition, mitochondria from KO sperm cannot form a proper mitochondrial sheath at the subsequent mitochondrial compaction step, although they can coil around the flagellum (Fig. 6*D*, *Lower Right*). To further understand the interrelationship of ARMC12 and TBC1D21, we generated *Tbc1d21* KO male mice carrying FLAG-tagged *Armc12* allele. We then studied ARMC12-VDAC2/3 interactions in the absence of TBC1D21 by co-IP experiments. Although the protein amount of FLAG-tagged ARMC12 in *Tbc1d21* KO TGC slightly decreased, the co-IP experiments demonstrated that ARMC12-VDAC2/3 interactions were disrupted in the absence of TBC1D21 (Fig. 6*E*). These results indicate that TBC1D21 is essential for the interactions of ARMC12 with VDAC2 and VDAC3. Thus, our genetic and proteomic studies disclosed that ARMC12 and TBC1D21 work cooperatively to target sperm mitochondria in the process of proper mitochondrial sheath formation.

## Discussion

To define the in vivo functions of the conserved and testis-enriched *Armc12* gene, we produced genetically engineered KO and FLAG-tagged KI mice. Our functional screens demonstrated that ARMC12 is essential for male fertility (Fig. 1*C*), and subsequent analysis revealed that ARMC12 is indispensable for proper mitochondrial elongation and sheath formation (Fig. 2*B*), the molecular mechanisms of which were previously unknown. While the JC1 data show that mitochondrial function is normal in *Armc12* KO spermatozoa (*SI Appendix*, Fig. S4*C*), the aberrant mitochondrial sheath formation in testis causes abnormal tail bending (Fig. 1*D*) and reduced sperm motility (Fig. 1*F*), impairing both sperm passage through the UTJ (Fig. 1*H*) and sperm-ZP binding (*SI Appendix*, Fig. S3 *B* and *C*). The direct cause of these impairments is thought to be the bending of the sperm tail which interrupts the *Armc12*-null sperm head from interacting with the UTJ epithelium and binding to the ZP. While the disruption of mitochondrial sheath formation was observed from spermatids in testis (Fig. 2*B*), the abnormal ratio of *Armc12*-null spermatozoa increased during epididymal migration (*SI Appendix*, Fig. S4*A*). This means that almost all KO spermatozoa have abnormalities in mitochondrial sheath formation during spermiogenesis, but not all spermatozoa in caput epididymis show prominent abnormalities such as tail bending. We do not have a clear reason for the increase in prominent abnormalities during sperm maturation, but a change of environment such as osmolality or physical stress caused by passing through the epididymis may be the reason for this increment. To summarize the sterility phenotype of *Armc12*-null mice: disruption of mitochondrial sheath formation due to the absence of ARMC12 leads to abnormal sperm-tail bending and reduced sperm motility, which causes impaired sperm passage through the UTJ and sperm-ZP binding, resulting in male infertility.

To uncover the molecular details of ARMC12 function, we performed in vitro analysis using cultured cells and found that overexpression of ARMC12 leads to clustering of mitochondria (Fig. 4*B* and Movie S5). These in vitro findings demonstrate that ARMC12 functions as an adherence factor between mitochondria and regulates mitochondrial dynamics. In vitro, ARMC12 interacts with TBC1D15 (*SI Appendix*, Fig. S6*G*), which is reported to be involved in the regulation of mitochondrial morphology in coordination with the mitochondrial transmembrane protein FIS1 (31, 32). Because overexpression of FIS1 leads to markedly reduced cristae (34), similar to our studies with ARMC12 overexpressed cells (Fig. 4*B*), ARMC12 could regulate mitochondrial dynamics via FIS1 and TBC1D15 (or their paralogs) in cultured cells. However, ARMC12-TBC1D15 interaction was not detected in vivo (Fig. 5*C*). In relation to this, ARMC12 is known to interact with nuclear protein RBBP4 to facilitate tumorigenesis of neuroblastoma (19). Thus, ARMC12 seems to interact with different proteins when it is expressed under nonphysiological conditions.

To further define the functions of ARMC12 in mitochondrial sheath formation, we searched for ARMC12-interacting proteins in vivo and discovered that ARMC12 binds to TBC1D21, GK2, MIC60, VDAC2, and VDAC3 in TGC (Fig. 5 *C* and *F*). TBC1D21 is expressed late in spermatids and localizes to the acrosome and midpiece (35), and the disruption of TBC1D21 causes male infertility with morphological abnormalities of the sperm mitochondria and flagellum in mice (33). However, because previous studies did not evaluate the mitochondrial sheath formation of *Tbc1d21*-null spermatids, we generated *Tbc1d21* KO mice and evaluated the formation. *Tbc1d21* KO male mice have an abnormality in mitochondrial sheath formation (Fig. 6*D*) similar to *Armc12* KO mice. However, because *Tbc1d21* KO sperm mitochondria can coil around the flagellum in an abnormal arrangement, ARMC12 and TBC1D21 have slightly different functions. Previous study revealed that *Gk2* KO male mice are sterile and have abnormalities in the arrangement of mitochondria at the interlocking step (3), the same step where ARMC12 functions; however, GK2 also acts differently from ARMC12 because *Gk2* KO sperm mitochondria can coil tightly around the flagellum in the subsequent mitochondrial compaction step. MIC60 is an abundant mitochondrial protein and is anchored in the inner mitochondrial membrane (36). Because MIC60 plays a crucial role in cristae junction formation and shaping inner membrane cristae (37), ARMC12 may contribute to cristae morphology by suppression of MIC60 in spermiogenesis. In fact, reduced cristae were observed in *Armc12*-overexpressing cells (Fig. 4*B*). Because ARMC12 is a mitochondrial peripheral membrane protein (Fig. 3*F*) and has no mitochondrial localization sequence, the mechanism by which ARMC12 localizes to mitochondria is an important point. ARMC1, which belongs to the same family of armadillo-repeat proteins as ARMC12, is also a mitochondrial peripheral membrane protein, similarly localizes to both the cytosol and mitochondria (14), and interacts with constituents of the mitochondrial contact site and cristae organizing system complex (38). In the present study, we reveal that ARMC12 interacts with the integral mitochondrial membrane proteins VDAC2 and VDAC3 (Fig. 5*F*), enabling ARMC12 to localize to the mitochondrial outer membrane. Because *Armc12* KO spermatids show abnormal mitochondrial elongation in the mitochondrial-interlocking step (Fig. 2*B*), ARMC12 is postulated to use VDAC2 and VDAC3 directly as scaffolds to link mitochondria in spermatids for proper mitochondrial elongation. To support the theory, *Vdac3* KO male mice are infertile and show abnormally shaped mitochondria in spermatids (39). ARMC12 interacts with several proteins including the mitochondrial inner/outer membrane proteins MIC60, VDAC2, and VDAC3 as well as testis-enriched proteins GK2 and TBC1D21 (Fig. 5 *C* and *F*). In addition, the presence of TBC1D21 is essential for mitochondrial sheath formation (Fig. 6*D*) and interactions of ARMC12 with VDAC2 and VDAC3 (Fig. 6*E*). These results indicate that ARMC12 plays a major role at the mitochondrial interlocking step through interactions with the VDAC proteins and works cooperatively with TBC1D21 on the sperm mitochondrial surface.

**Fig. 7.**
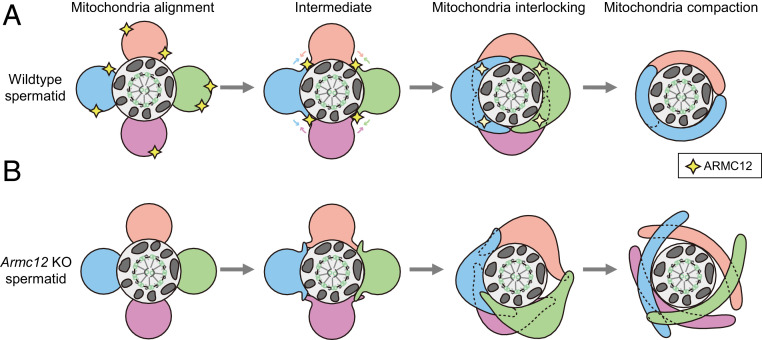
Schematic models for the function of ARMC12. (*A*) Schematic model of mitochondrial sheath formation observed in WT spermatids. (*B*) Schematic model of abnormal mitochondrial sheath formation observed in *Armc12* KO spermatids.

To make a well-arranged mitochondrial sheath, sperm mitochondria change their morphology during spermiogenesis. Spherical mitochondria form four helical arrays (mitochondria alignment), and each mitochondrion elongates laterally in two directions to reach the opposite side of the mitochondria between the mitochondrial-alignment and mitochondrial-interlocking steps (2, 8). Mitochondrial elongation during mitochondrial sheath formation starts with the emergence of mitochondrial tips late in the mitochondrial-alignment step (Fig. 7*A*). Elongating ends of normal sperm mitochondria are hidden under the adjacent mitochondria in the mitochondrial-interlocking step, but several mitochondrial tips were observed in *Armc12* KO spermatids (Fig. 2*B*), implying that each mitochondrion elongates in the free direction and that ARMC12 functions as a guide for the proper mitochondrial elongation (Fig. 7*A*). To function as a guide for mitochondrial elongation, we propose that ARMC12 tethers adjacent mitochondria using VDAC2 and VDAC3 as scaffolds. In the absence of ARMC12, an aberrant elongation of sperm mitochondria causes the disruption of the interlocking structure and subsequent uncoiled sperm mitochondria around the flagellum (Fig. 7*B*). In summary, ARMC12 regulates spatiotemporal mitochondrial dynamics through cooperative interactions with several proteins on the mitochondrial surface during mitochondrial sheath formation.

*ARMC12* shows testis-enriched expression both mice and humans (Fig. 1 *A* and *B*). To identify the *ARMC12*-expressing spermatogenic cell types in humans, we analyzed the *ARMC12*-expressing cell population using single-cell RNA data (40). Single-cell RNA data revealed that *ARMC12* is expressed strongly in spermatids but not in other cells in both mice and humans (*SI Appendix*, Fig. S9 *A* and *B*). In addition, the down-regulation of the *ARMC12* transcript is observed in infertile men with maturation arrest, oligospermia, and Sertoli cell-only syndrome (41). These data imply that the highly conserved (*SI Appendix*, Fig. S1*A*) and spermatid-restricted human ARMC12 protein functions in mitochondrial sheath formation as well as mice. Thus, our findings will contribute to a treatment for infertile men with abnormal mitochondrial sheath formation and development of male contraceptives. Our studies have uncovered major clues in elucidating “sperm mitochondrial dynamics” that are regulated by ARMC12 during spermiogenesis, and the genetic models that we have created will continue to reveal the molecular mechanisms of mitochondrial sheath formation.

## Materials and Methods

### Animals.

All animal experiments were approved by the Animal Care and Use Committee of the Research Institute for Microbial Diseases, Osaka University. To generate gene-manipulated mice, we used the CRISPR/Cas9 system (28, 42, 43). More detailed information is provided in *SI Appendix*, *Supplementary Materials and Methods*. The IDs of gene-manipulated lines used in this study are summarized in *SI Appendix*, Table S1.

### RT-PCR.

Using TRIzol reagent (Thermo Fisher Scientific), total RNA was isolated from multiple adult tissues of C57BL6/129S5 hybrid mice, testes of 5- to 42-d-old mice, and multiple adult human tissues obtained from the Human Tissue Acquisition and Pathology core service (Baylor College of Medicine). Human tissue acquisition was approved by the Institutional Review Board of Baylor College of Medicine. All human samples were de-identified and informed consent was obtained prior to use. Mouse and human cDNA were prepared using SuperScript III Reverse Transcriptase (Thermo) following the manufacturer’s instructions. PCR was performed using Taq DNA polymerase with ThermoPol buffer (New England Biolabs). The primers and amplification conditions for each gene are summarized in *SI Appendix*, Table S2.

### Fertility Analysis of KO Mice.

To confirm the fertility of KO male mice, natural mating tests were conducted. Three male mice were individually caged with two or three B6D2F1 females for 2 mo. Both plug and pup numbers were checked at ∼10 AM every weekday to determine the number of copulations and litter size.

### Phenotypic Analysis of KO Mice.

Phenotypic analysis of KO mice was performed as written in *SI Appendix*, *Supplementary Materials and Methods*. The antibodies used in this study are listed in *SI Appendix*, Table S3.

### Sperm Motility Analysis.

Sperm motility analysis was performed as previously described (44). Cauda epididymal spermatozoa were suspended and incubated in Toyoda, Yokoyama and Hoshi (TYH) medium that can induce sperm capacitation (45). Sperm motility was then measured using the CEROS sperm analysis system (software version 12.3; Hamilton Thorne Biosciences). Analysis settings were as previously described (46). The motility of epididymal spermatozoa was recorded after 30 min and 2 h of incubation in TYH medium. Movies of control and *Armc12* KO spermatozoa were recorded after 30 min incubation in TYH as previously described (44).

### Ultrastructural Analyses of Testes.

Ultrastructural analyses of testes using TEM and SEM were conducted as previously described (3).

### Ultrastructural Analysis of Spermatozoa Collected from Cauda Epididymis.

Cauda epididymal spermatozoa were incubated in TYH medium to disperse. Spermatozoa were collected into a 2.0-mL tube and washed with 0.1 M phosphate buffer (pH 7.4). Spermatozoa were mounted on coverslips and fixed with 1% glutaraldehyde in 0.1 M phosphate buffer on ice. After washing, the specimens were postfixed with 1% osmium tetroxide in 0.1 M phosphate buffer containing 1% potassium ferrocyanide and conductive-stained with 1% tannic acid solution and 1% osmium tetroxide solution. The specimens were dehydrated in graded series of ethanol and then critical point dried using a Samdri-PVT-3D system (Tousimis). The specimens were coated with osmium tetroxide using osmium coater HPC-30W (Vacuum Device). Electron micrographs were captured with S-4800 field emission scanning electron microscope (Hitachi).

### In Vitro Analysis of ARMC12.

Descriptions of in vitro analysis performed in this study are described in *SI Appendix*, *Supplementary Materials and Methods*.

### Statistical Analyses.

Statistical analyses were performed using a two-tailed unpaired *t* test (*n* ≥ 3) by GraphPad Prism 6 (GraphPad). *P* values less than 0.05 were considered significant. Data represent the means and error bars indicate SD.

## Supplementary Material

Supplementary File

Supplementary File

Supplementary File

Supplementary File

Supplementary File

Supplementary File

## Data Availability

All study data are included in the article and/or supporting information.
