# Supplementary material for: ARMC12 regulates spatiotemporal mitochondrial dynamics during spermiogenesis and is required for male fertility
Source: Proc Natl Acad Sci U S A. 2021 Feb 3;118(6):e2018355118. doi: 10.1073/pnas.2018355118 (PMC8017931; doi:10.1073/pnas.2018355118)
Supplement: Supplementary File [file pnas.2018355118.sapp.pdf]

PNAS
www.pnas.org

Supplementary Information for

ARMC12 regulates spatiotemporal mitochondrial dynamics during spermiogenesis and is required for male fertility

Keisuke Shimada[a], Soojin Park[a,b], Haruhiko Miyata[a], Zhifeng Yu[c,d], Akane Morohoshi[a,b], Seiya Oura[a,e], Martin M. Matzuk[c,d,*] and Masahito Ikawa[a,b,e,f,*]

[a] Research Institute for Microbial Diseases, Osaka University, Osaka, 5650871, Japan
[b] Graduate School of Medicine, Osaka University, Osaka, 5650871, Japan
[c] Center for Drug Discovery, Baylor College of Medicine, Houston, TX 77030, USA
[d] Department of Pathology & Immunology, Baylor College of Medicine, Houston, TX 77030, USA
[e] Graduate School of Pharmaceutical Sciences, Osaka University, Osaka, 5650871, Japan
[f] The Institute of Medical Science, The University of Tokyo, Minato-ku, Tokyo, 1088639, Japan
[*]To whom correspondence should be addressed.

Email: mmatzuk@bcm.edu (M.M.M) or ikawa@biken.osaka-u.ac.jp (M.I)

This PDF file includes:

Supplementary text
Figures S1 to S9
Tables S1 to S3
Legends for Movies S1 to S5
SI References

Other supplementary materials for this manuscript include the following:

Movies S1 to S5

Supplementary Text

Supplementary Materials and Methods

Animals. All animal experiments were approved by the Animal Care and Use committee of the Research Institute for Microbial Diseases, Osaka University. Animals were housed in a temperature-controlled environment with 12 h light cycles and free access to food and water. B6D2F1 (C57BL/6 × DBA2) mice, ICR mice or C57BL6/J were used as embryo donors, foster mothers or gene cloning, respectively. The C57BL6/129S5 hybrid mice were used to collect RNA for RT-PCR. B6D2-Tg(CAG/Su9-DsRed2, Acr3-EGFP)RBGS002Osb (RBGS, Red Body Green Sperm) mice (1) were used for observing sperm mitochondria.

Gene-manipulated mouse lines used in this study were deposited at both the Riken BioResource Center (Riken BRC; Tsukuba, Japan) and the Center for Animal Resources and Development, Kumamoto University (CARD; Kumamoto, Japan). All lines are available through these centers. The ID of each line is summarized in SI Appendix, Table S1.

Generation of *Armc12* Indel KO Mice. To generate the *Armc12* KO mice, we prepared the pX330 plasmid (#42230, Addgene, Cambridge, MA, USA) expressing a chimeric guide RNA together with human codon-optimized Cas9 (hCas9). The cleavage target site was designed in exon 1 directly downstream of the start codon of *Armc12* (SI Appendix, Fig. S2A), and the pX330 plasmid that expressed a single-guide RNA and humanized CAS9 protein was constructed. For avoiding off-target cleavage, we checked the specificity of the guide RNA sequences with a homology search using Bowtie (2). Plasmid DNA for injection was purified from bacterial colonies using a NucleoBond Xtra Midi kit (Macherey-Nagel, Düren, Germany), and Sanger sequenced using the primer (5'-TGGACTATCATATGCTTACC-3'). Before injection, the DNA cleavage activity of the plasmid was checked using the HEK 293T EGFP assay (3). *Armc12* indel KO mice were generated by microinjecting 5.0 ng/μL of pX330 plasmid into oocytes. Eggs were cultured in KSOM medium (4) overnight and then transferred into the oviducts of pseudopregnant ICR females. Pups were obtained by C-section followed by fostering onto lactating mothers. Tail biopsies were performed for isolating genomic DNA for genotyping. Of the 153 fertilized oocytes that were injected, 91 eggs were transplanted into the oviducts of pseudopregnant females. A total of 14 founder mice (F0) were born, and 4 pups possessed a mutation. *Armc12* mutant mice were maintained by sibling cross. *Armc12* indel KO mice were mated with RBGS mice for generating *Armc12* indel KO mice expressing both EGFP in the acrosome and DsRed2 in the mitochondria (*Armc12* indel KO mice with RBGS).

Generation of *Armc12* large-deletion KO Mice. *Armc12* large deletion (LD) mice were generated by electroporation described previously (5). The cleavage target sites were designed after the start codon and in a site overlapping the stop codon of *Armc12* (SI Appendix, Fig. S5A). The crRNA sequences used in this study were 5'-TCCCCGGTTCCTGGAGCAAC-3' and 5'-AAAACCGGGAAGTGAAGTTA-3' (SI Appendix, Fig. S5A). Synthesized crRNAs (Merck, Darmstadt, Germany), tracrRNA (Merck) and CAS9 protein (Thermo Fisher Scientific, Waltham, MA, USA) were incubated to make the CAS9 ribonucleoprotein (RNP) complex. The obtained complex was electroporated into fertilized oocytes using NEPA21 electroporator (NEPA GENE, Chiba, Japan). Of the 80 fertilized oocytes that had been electroporated, 78 eggs were transplanted into the oviducts of pseudopregnant females. A total of 18 founder mice (F0) were born, and 3 pups possessed mutations. Genotyping and generation of *Armc12* large deletion KO mice with RBGS were conducted as described above.

Generation of FLAG-tagged *Armc12* Knock-in Mice. FLAG-tagged *Armc12* KI mice were generated as previously described (6). We designed a guide RNA that recognizes sequences close to the stop codon (Fig 3A) and inserted the sequence into the pX459 V2.0 plasmid (#62988, Addgene). The reference plasmids were constructed in pBluescript II SK (+) vector (Agilent, Palo Alto, CA, USA) using In-Fusion HD cloning kit (Clontech, Mountain View, CA, USA). The EGR-G01 embryonic stem (ES) cells (7) were co-transfected with 1.0 μg of guide RNA inserted vector and 1.0 μg of reference plasmid. Seven ES clones out of 24 had the expected KI allele. ES cell clones that possessed the proper KI allele were injected into ICR embryos and chimeric

blastocysts were transferred into the uteri of pseudopregnant females. Generated chimeric male mice were mated with B6D2F1 female mice to obtain the FLAG-tagged *Armc12* KI heterozygous mice.

Generation of *Tbc1d21* KO Mice. *Tbc1d21* KO mice were generated using methodology as previously described (6). In brief, we designed two guide RNAs that recognizes regions upstream of the start codon and downstream of the stop codon (Fig 6A) and inserted the sequence into pX459 V2.0 plasmid. The EGR-G01 ES cells were co-transfected with two guide RNAs to generate *Tbc1d21* KO ES cells. ES clones were injected into ICR embryos and chimeric blastocysts were transferred into the uteri of pseudopregnant females to produce chimeric offspring. High percentage chimeric male mice were mated with B6D2F1 female mice to obtain heterozygous male and female mice carrying the *Tbc1d21* large deletion.

Sequence Comparison Analysis. Amino acid sequences of ARMC12 were obtained from the NCBI Entrez Protein database. Clustal W2.1 was used for multiple sequence alignment (8). The accession numbers of the ARMC12 proteins used in this study were as follows: turtle (XP_008174027.2), opossum (XP_007483820.1), dog (XP_532120.02), mouse (NP_080566.2) and human (NP_001273503.1).

Genotype Analysis. Polymerase chain reaction (PCR) was performed using KOD FX neo (Toyobo). The primers and amplification conditions for each gene are summarized in SI Appendix, Table S2. For checking the DNA sequence, PCR products were purified using a Wizard SV Gel and PCR Clean-Up System (Promega, Madison, WI, USA) kit, and Sanger sequenced with an ABI 3130*xl* Genetic Analyzer (Thermo) using sequence primer. All oligonucleotides were purchased from GeneDesign (Osaka, Japan).

Morphological and Histological Analysis of Testis. Male mice (11-12 weeks old) were euthanized and testes were dissected. After measuring the testicular weight, testes were fixed with Bouin's fixative (Polysciences, Warrington, PA, USA). Fixed testes were embedded in paraffin, sectioned, rehydrated, and treated with 1% periodic acid for 10 min, followed by treatment with Schiff's reagent (Wako, Osaka, Japan) for 20 min. The sections were stained with Mayer's haematoxylin solution prior to imaging and observed using a BZ-X710 microscope (Keyence, Osaka, Japan).

Morphological Analysis of Spermatozoa. Spermatozoa from male mice with RBGS transgenes were used for this analysis. Spermatozoa were collected from the cauda epididymis, and suspended in TYH medium (9) with 10 µg/mL of Hoechst 33342 (Thermo). After a 10 min incubation at 37 °C under 5% CO_2, a sperm suspension was mounted on MAS coated glass slide (Matsunami Glass, Osaka, Japan), and a cover slip (Matsunami Glass) was added. Immunofluorescence and sperm shape were observed using a Nikon Eclipse Ti microscope connected to a Nikon C2 confocal module (Nikon, Tokyo, Japan).

Morphological analysis of spermatozoa collected from the three regions of the epididymis were conducted as previously described (10).

***In vitro* Fertilization and Zona Pellucida Binding Assay.** *In vitro* fertilization (IVF) was performed as previously described (10). Cumulus-intact, cumulus-free, and zona-free oocytes were used for IVF. To assess the zona pellucida binding ability of control and *Armc12* KO spermatozoa, cumulus-free oocytes were incubated with 2.0×10^5 sperm/mL capacitated sperm for 30 min and fixed with 4% paraformaldehyde (PFA) (11). The percentage of oocytes with attached spermatozoa was calculated using a BX50 DIC microscope (Olympus, Tokyo, Japan).

Imaging of Sperm Inside the Female Reproductive Tract. Live imaging of spermatozoa inside the female reproductive tract was conducted as previously described (1). B6D2F1 female mice more than 8 weeks old were superovulated and mated with WT with RBGS transgenes or *Armc12* KO male mice with RBGS transgenes 12-14 h after hCG injection. Vaginal plugs were checked every 30 min, and female mice were sacrificed 2-3 h after observing a vaginal plug.

Female reproductive tracts were dissected and spermatozoa inside the reproductive tracts were observed using a BZ-X710 microscope. Fluorescent images were false-colored using ImageJ software (v 1.52, NIH, Bethesda, MD, USA).

Immunoblot Analysis. Immunoblot analysis was conducted as previously described (10) with slight modifications. Cell lysates from HEK293T cells were obtained using a Pierce IP Lysis Buffer (Thermo). Proteins from testis or spermatozoa were extracted using a T-PER Tissue Protein Extraction Reagent (Thermo). Proteins were separated by SDS-PAGE under reducing conditions and transferred to polyvinylidene fluoride (PVDF) membrane using the Trans Blot Turbo system (Bio-Rad, Munich, Germany). After blocking with 10% skim milk (Becton Dickinson, Cockeysville, MD, USA), the membrane was incubated with primary antibody overnight at 4 °C, and then incubated with 1:5000 dilution of HRP-conjugated secondary antibody for 2 h at room temperature. Chemiluminescence was detected by Chemi-Lumi One Super (Nacalai Tesque, Kyoto, Japan) using the Image Quant LAS 4000 mini (GE Healthcare, Chicago, IL, USA). The antibodies used in this study are listed in SI Appendix, Table S3.

Evaluation of Mitochondrial Activity of Spermatozoa Using JC-1. Spermatozoa were collected from the mouse cauda epididymis, and mitochondrial activity was checked by JC-1 (5,5',6,6'-tetrachloro-1,1',3,3'-tetraethylbenzimidazolylcarbocyanine iodide, Thermo) (12). In the present study, we used FHM (13) that does not contain pyruvate for medium. Cauda epididymal spermatozoa were incubated with the medium, and JC-1 reagent was added (5.0 μM, final concentration). The mixture was incubated in the dark at 37 °C for 20 min. After the incubation, sperm was mounted on MAS coated glass slide with a cover slip and observed by an Olympus BX53 differential interference contrast microscope equipped with an Olympus DP74 color camera. The fluorescence of JC-1 monomer and JC-1 multimer were taken by U-FBNA fluorescence filter cube and U-FGW fluorescence filter cube (Olympus), respectively. Quantitative analysis was performed using ImageJ software. The fluorescence ratio was obtained by dividing quantified orange fluorescence by quantified green fluorescence of each sperm midpiece.

Phase Separation of Testicular Extracts Using Triton X-114. Phase separation of testicular extracts was conducted as previously described with slight modification (14). Testes were dipped in PBS (-), placed on ice, and then sonicated with a sonicator (SLPe, Branson Ultrasonics, Danbury, CT, USA). Sonicated samples were incubated 1 h on ice with occasional vortexing. After centrifuging at 15,000 rpm for 30 min at 4 °C, the supernatants were collected in microtubes as an aqueous phase. After removing the inner layer, PBS (-) was added to the pellet and resuspended (detergent phase). Both aqueous and detergent phases were mixed with SDS-sample buffer, boiled and subjected to SDS-PAGE and immunoblot analysis. COX IV was used for positive control of membrane-protein, and ZPBP was used for soluble-protein (15, 16). The antibodies used in this study are listed in SI Appendix, Table S3.

Removal of Peripheral Proteins with High Salt Treatment. Removal of peripheral proteins was performed as previously described (17) with slight modification. Mitochondria were isolated from FLAG-tagged *Armc12* Ki testis using a mitochondria isolation kit for tissue (Thermo) following the manufacturer's instructions. Isolated mitochondria were suspended in isotonic buffer (50 mM Tris-HCl (pH 8.0), 50 mM KCl, 0.2 mM EDTA) or 1 M NaCl (high salt) in isotonic buffer. Each of the buffers contained 1:100 dilution protease inhibitor cocktail (Nacalai). After incubation of the sample on a rotating wheel at 4 °C for 60 min, the samples were recovered by centrifugation. Both pellet and supernatant were analyzed.

Immunofluorescence of Testes and Spermatozoa. Immunofluorescence analysis of testes was performed using cryosections as previously described (18). Rabbit anti-FLAG (PM020) 1:200 was used for primary antibody and AlexaFluor 546 anti-rabbit 1:500 (Thermo) was used for secondary antibody. The stage of the cycle was identified based on the morphological characteristics of the spermatids, in particular their nucleus (19). Fluorescent images were false-colored and cropped using ImageJ software.

Immunofluorescence analysis of spermatozoa using IZUMO1 antibody (KS64-125) was performed as previously described (20) with slight modification. After 2 h of incubation in TYH drops, spermatozoa were smeared on microscope slides, fixed with 4% PFA, blocked with 3% BSA for 1 h and immunostained with anti-IZUMO1 antibody (1:1000). Goat-anti rat Alexa Fluor 488 (1:250) was used as the secondary antibody.

Immunoelectron Microscopy Using Testicular Sections. Testes were dissected after perfusion fixation with 4% PFA in PBS under anesthesia and sliced into 2 mm thick sections. Sectioned samples were fixed with 4 % formaldehyde in 0.1M phosphate buffer (pH 7.4) and washed with 0.1M phosphate buffer (pH7.4) containing 4% sucrose. For cryo-protection, tissue slices were incubated in 10%, 15%, and 20% sucrose in 0.1 M phosphate buffer (pH 7.4) for 6 h each, embedded in OCT compound (Sakura, Tokyo, Japan), and frozen in liquid nitrogen. Six-micrometer-thick sections were cut at -20 °C using a cryostat (Thermo), and the cryo-sections were attached to MAS coated glass coverslips (Matsunami Glass) and air-dried for 30 min.

The samples were blocked with blocking solution (0.1M phosphate buffer containing 0.1% saponin, 10% BSA, 10% normal goat serum and 0.1% cold water fish skin gelatin) for 30 min. The blocking solution was replaced with mouse anti-FLAG (M2) antibody 1:150 in blocking solution and samples were incubated overnight at 4 °C. The sections were washed with 0.1M phosphate buffer (pH 7.4) containing 0.005% saponin. Samples were incubated with goat anti-mouse IgG coupled to 1.4 nm gold 1:300 (Nanogold, Nanoprobes, Yaphank, NY, USA) in blocking solution as a secondary antibody for 3 h. The samples were washed with 0.1 M phosphate buffer (pH 7.4) containing 0.005% saponin, followed by washing in 0.1 M phosphate buffer (pH 7.4), and then fixed with 1% glutaraldehyde in 0.1 M phosphate buffer (pH 7.4) for 10 min. The sections were washed in PBS containing 50 mM glycine, followed by washing in PBS containing 1% BSA in water. Gold labeling was intensified with GoldEnhance EM kit (Nanoprobes) for 3 min. The gold intensification solution was removed, the sections were soaked in 1% sodium thiosulfate solution for a few seconds, and washed in water. The sections were post-fixed in 1% OsO_4 and 1.5% potassium ferrocyanide in 0.1 M phosphate buffer (pH 7.4) for 1 h. Samples were dehydrated in a graded series of ethanol, substituted with propylene oxide, and embedded in epoxy resin. Eighty nm ultrathin sections were stained with 8% uranyl acetate and lead staining solution. The samples were examined using a JEM-1400 plus electron microscope (JEOL, Tokyo, Japan) at 80 kV with a CCD Veleta 2K × 2K camera (Olympus).

Generation of Tagged ARMC12 Expressing Vector. Mouse *Armc12* cDNA (1023 bp) was cloned from the cDNA of C57BL/6J mouse testis by PCR and inserted into FLAG-tagged pCAG vectors that contain the CAG promoter and a rabbit globin poly(A) signal (21). For generating truncated ARMC12 vectors, invers PCR was conducted using a FLAG-tagged pCAG ARMC12 vector as a template. Truncated ARMC12 vectors were generated using this amplicon by a KOD plus mutagenesis kit (Toyobo) following the manufacturer's instructions. The primers used in this study are listed in SI Appendix, Table S2.

Immunofluorescence and Time Lapse Imaging of Mitochondria in COS-7 cells. COS-7 cells (1.0×10^5 cells) were seeded on cover slips in 6-well plate. After 6-8 h, *Armc12* expressing vectors were transfected into COS-7 cells using Lipofectamine LTX (Thermo). After 24 h, cells were fixed by 4% PFA and permeabilized with 0.5% Triton X-100. Cells were blocked with 3% bovine serum albumin (BSA) and immunostained. Rabbit anti-FLAG (F7425, Merck) and mouse anti-TOM20 (F-10, Santa Cruz) were used for primary antibody. The cells were incubated with these antibodies overnight at 4 °C. After washing three times, the cells were incubated with both AlexaFluor 488 anti-rabbit and AlexaFluor 546 anti-mouse antibodies for 2 h at room temperature. The cells were then washed three times and stained with Hoechst 33342 for visualizing nuclei. After washing three times, the cells were mounted on MAS coated glass slides. Immunofluorescence was observed using a Nikon Eclipse Ti microscope connected to a Nikon C2 confocal module.

For analysis of mitochondrial behavior in COS-7 cells after FLAG-tagged *Armc12* overexpression, time lapse series were obtained at 3-min intervals. COS-7 cells were seeded on coverglass chamber (AGC Techno Glass, Shizuoka, Japan). After 6 h, FLAG-tagged *Armc12*

expressing vectors were transfected into COS-7 cells using Lipofectamine LTX and 200 nM Mito tracker Red FM (Thermo) was added on medium 12 h after transfection. Mitochondrial behavior was observed from 15 h after transfection using a BZ-X710 microscope.

Ultrastructural Analysis of COS-7 Cells Using TEM. COS-7 cells (2.0× 10^5 cells) were cultured on a polystyrene cover slip (Cell Desk, Sumitomo Bakelite, Tokyo, Japan). After 6-8 h, *Armc12* expressing vector was transfected into COS-7 cells using Lipofectamine LTX. The cells were fixed with 4% PFA in 0.1M phosphate buffer (pH7.4) 24 h after transfection. Cells were washed three times in the same buffer, post-fixed for 1 h with 1% osmium tetroxide and 0.8% potassium ferrocyanide in 0.1 M phosphate buffer (pH7.4), dehydrated in graded series of ethanol and embedded in Epon812 (TAAB, Berkshire, UK). Eighty nm ultra-thin sections were stained with saturated uranyl acetate and lead citrate solution. The samples were examined using a JEM-1400plus electron microscope (JEOL) at 80 kV with a CCD Veleta 2K × 2K camera (Olympus).

Immunoelectron Microscopy Using Cell Culture. COS-7 cells (2.0× 10^5 cells) were cultured on polystyrene cover slips (Cell Desk, Sumitomo Bakelite, Tokyo, Japan). After 6-8 h, *Armc12* expressing vector was transfected into COS-7 cells using Lipofectamine LTX. Cells were fixed with 4% PFA in 0.1M phosphate buffer (pH7.4) 24 h after transfection. The cells were washed three times with 0.1M phosphate buffer (pH7.4) containing 4% sucrose. The samples were blocked, immunolabeled with mouse anti-FLAG (M2) antibody, cut into ultrathin sections, and observed as described in immunoelectron microscopy using testicular sections.

Mass Spectrometry Analysis Using the BioID2 System. Coding sequence of *Armc12* was inserted into MCS-BioID2-HA (#74224, Addgene) to generate a Biotin ligase (BirA)-fused ARMC12 plasmid. Mock or BirA-fused ARMC12 plasmid were transfected into HEK293T cells with 50 μM biotin. Cell lysates were obtained 24 h after transfection using Pierce IP Lysis Buffer, and biotinylated proteins were pulled down as previously described (22). Biotinylated proteins were eluted using an SDS-PAGE sample buffer, boiled at 95 °C for 5 min, and then applied to the SDS-PAGE under reducing conditions. The proteins on gel were visualized by the Rapid Stain CBB Kit (Nacalai Tesque), and excised.

The proteins were reduced with 10 mM dithiothreitol (DTT), followed by alkylation with 55 mM iodoacetamide, and digested in-gel by treatment with trypsin and purified with C18 tip (GL-Science, Tokyo, Japan). The resultant peptides were subjected to nanocapillary reversed-phase LC-MS/MS analysis using a C18 column (25 cm × 75 um, 1.6 μm; IonOpticks, Victoria, Australia) on a nanoLC system (Bruker Daltoniks, Bremen, Germany) connected to a timsTOF Pro mass spectrometer (Bruker Daltoniks) and a modified nano-electrospray ion source (CaptiveSpray; Bruker Daltoniks). The mobile phase consisted of water containing 0.1% formic acid (solvent A) and acetonitrile containing 0.1% formic acid (solvent B). Linear gradient elution was carried out from 2% to 35% solvent B for 18 min at a flow rate of 400 nL/min. The ion spray voltage was set at 1.6 kV in the positive ion mode. Ions were collected in the trapped ion mobility spectrometry (TIMS) device over 100 ms and MS and MS/MS data were acquired over an *m/z* range of 100-1,700. During the collection of MS/MS data, the TIMS cycle was adjusted to 1.1 s and included 1 MS plus 10 parallel accumulation serial fragmentation (PASEF)-MS/MS scans, each containing on average 12 MS/MS spectra (>100 Hz) (23), and nitrogen gas was used as collision gas.

The resulting data was processed using DataAnalysis version 5.1 (Bruker Daltoniks), and proteins were identified using MASCOT version 2.6.2 (Matrix Science, London, UK) against the Swiss-Prot database. Quantitative value and fold exchange were calculated by Scaffold4 (Proteome Software, Portland, OR, USA) for MS/MS-based proteomic studies (24).

Immunoprecipitation. Cell lysates from HEK293T cells or mouse testis were obtained using a Pierce IP Lysis Buffer (Thermo) or T-PER Tissue Protein Extraction Reagent (Thermo), respectively, following the manufacturer's instructions. Solubilized proteins were mixed with Dynabeads Protein G (Thermo)-conjugated with 2.0 μg of antibody. The immune complexes were incubated for 1 h at 4 °C and co-immunoprecipitated (co-IP) products were eluted with SDS-

sample buffer and denatured for 10 min at 70 °C. The antibodies used in this study are listed in SI Appendix, Table S3.

MS Analysis Using Isolated Mitochondria from Testes. Mitochondrial isolation from testes was performed using a mitochondria isolation kit for tissue (Thermo) following the manufacturer's instructions. Protein lysates were obtained from isolated mitochondria using T-PER Tissue Protein Extraction Reagent. Immunoprecipitation was performed as described above using an anti-FLAG antibody (clone FLA-1, MBL, Aichi, Japan), and the immunoprecipitated proteins were obtained by FLAG peptide elution. Eluted proteins were then subjected to MS analysis as described above.

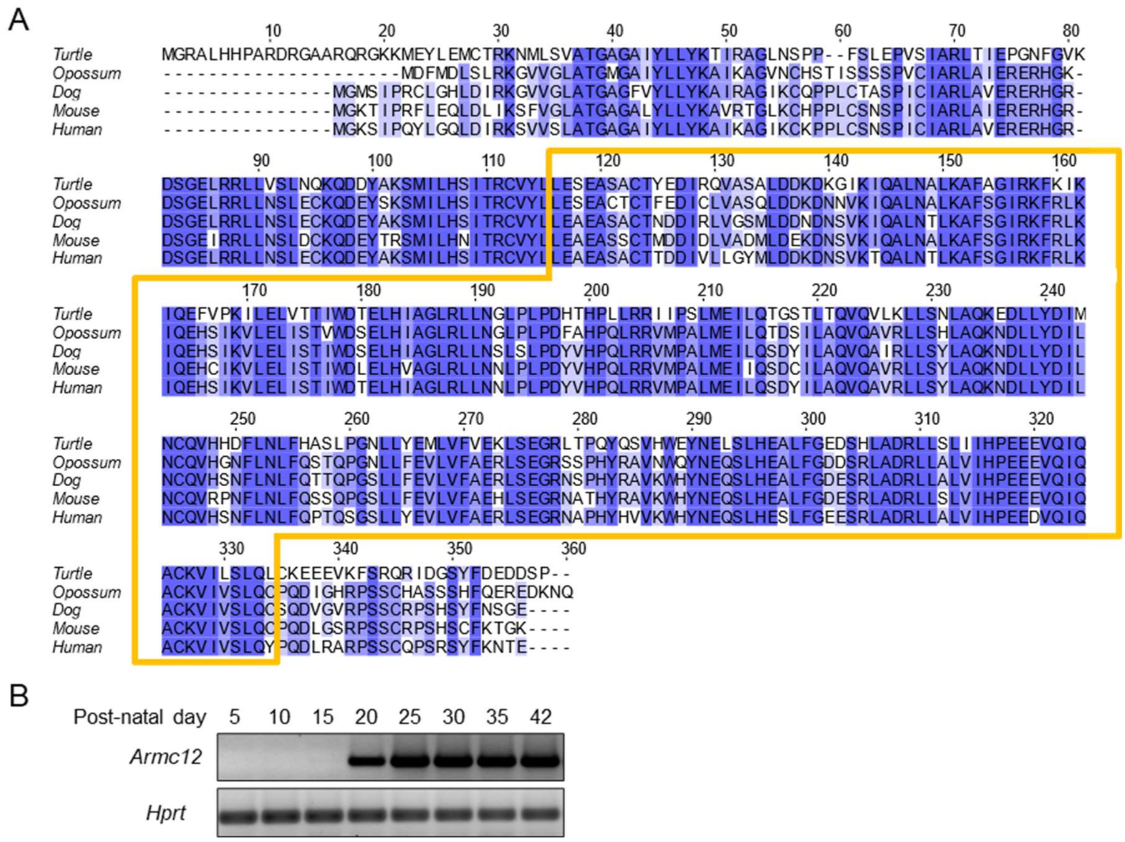

Fig. S1. ARMC12 is conserved among reptiles, marsupials and mammals. (*A*) Sequence alignment of ARMC12 proteins from several species. Orange box indicates the ARM domain. (*B*) RT-PCR for *Armc12* from mouse testes at various postnatal days with *Hprt* as a control.

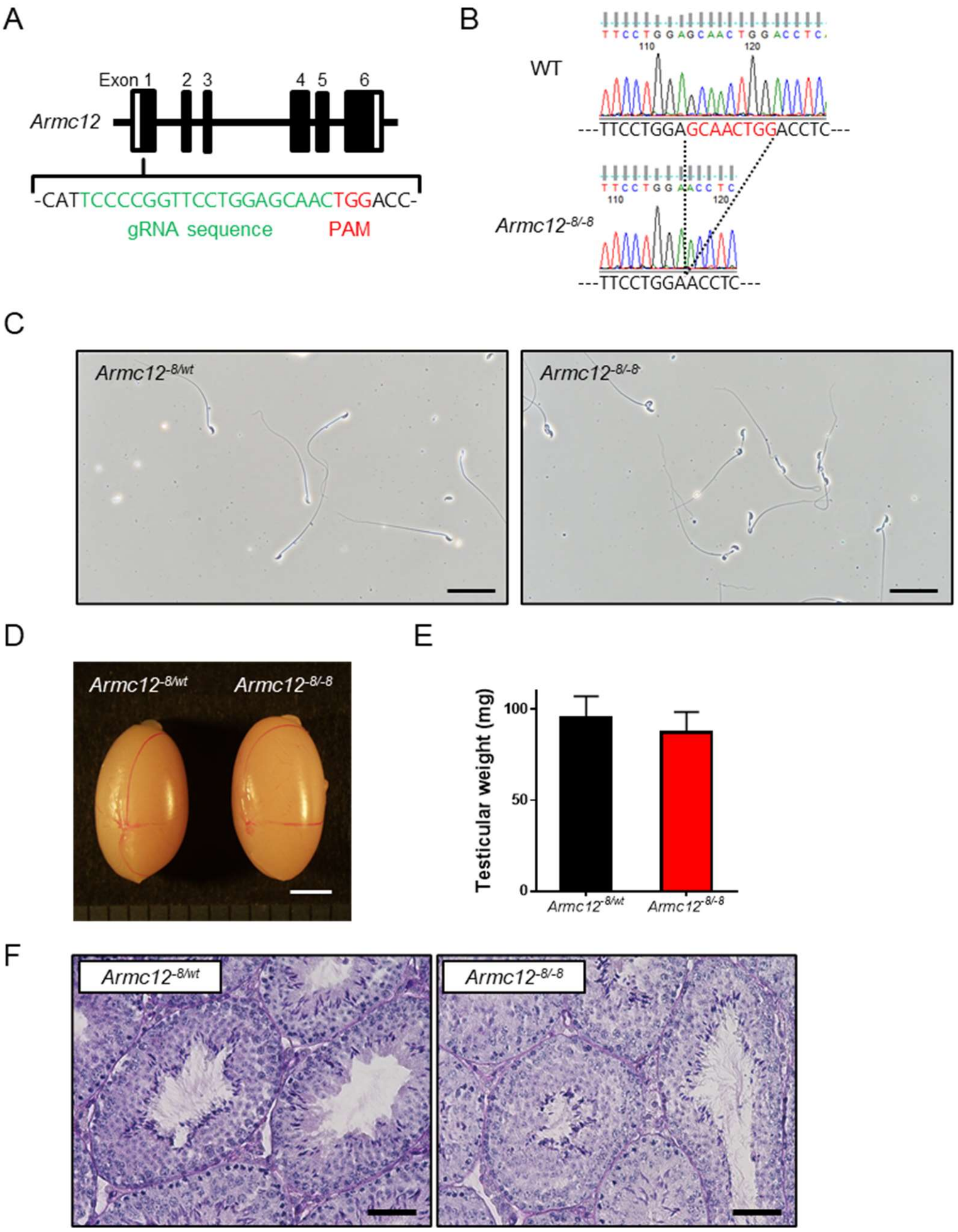

Fig. S2. ARMC12 is conserved among reptiles, marsupials and mammals. (*A*) Design of guide RNA for generating *Armc12* indel KO mice. Guide sequence is highlighted in green and PAM motif is highlighted in red, respectively. (*B*) Control and *Armc12*$^{-8/-8}$ alleles. Red letters indicate 8 bp deletion site. (*C*) Sperm morphology of control and *Armc12* indel KO mice. Scale bars are 50 µm. (*D*) Gross morphology of adult control and *Armc12* indel KO testes. Scale bar is 2 mm. (*E*) Average weight of adult control and *Armc12* indel KO testis from 11-12 week old mice (*n*=5). Error bars represent S.D. (*F*) PAS staining of testis sampled from control and *Armc12* indel KO male mice. Scale bars are 50 µm.

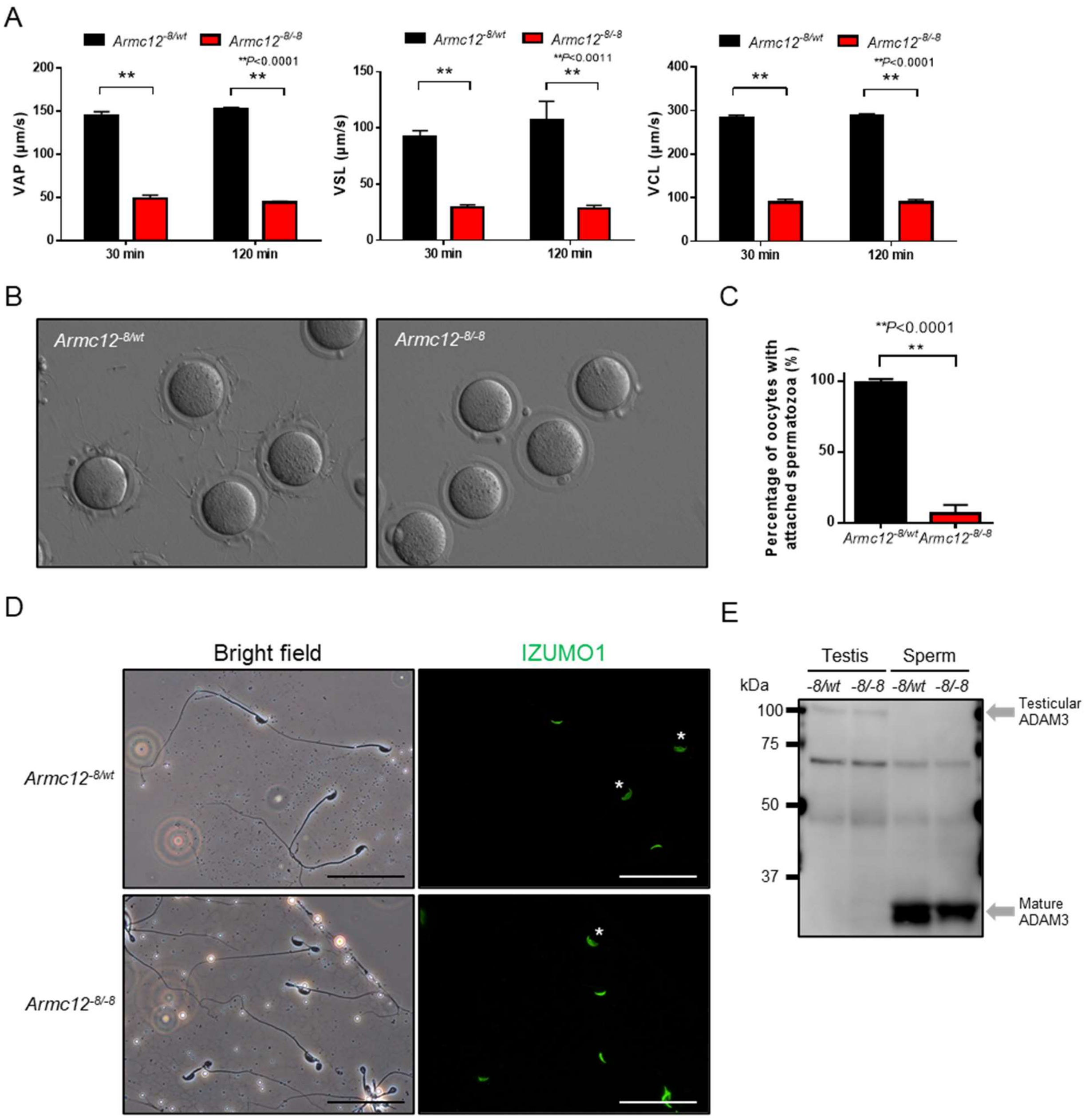

Fig. S3. *Armc12* indel KO mice show decreased sperm motility, and null spermatozoa have a defect in sperm-ZP binding. (*A*) Sperm motility parameters are shown. Mean ± S.D. for: VAP, average path velocity; VSL, straight-line velocity; and VCL, curvilinear velocity. ** P<0.01, Student's t test (n=3). (*B*) Sperm-ZP binding assay *in vitro*. Oocytes were inseminated 30 min with control or *Armc12* indel KO spermatozoa, and then observed. (*C*) Percentage of oocytes with attached spermatozoa 30 min after sperm-ZP binding assay. ** P<0.01, Student's t test, error bars represent S.D. (n=4). (*D*) Immunostaining of IZUMO1 in spermatozoa before and after the acrosome reaction. Asterisks mark acrosome reacted spermatozoa. Scale bars are 50 µm. (*E*) WB analysis of ADAM3 in both testis and cauda epididymal spermatozoa from control and *Armc12* indel KO mice.

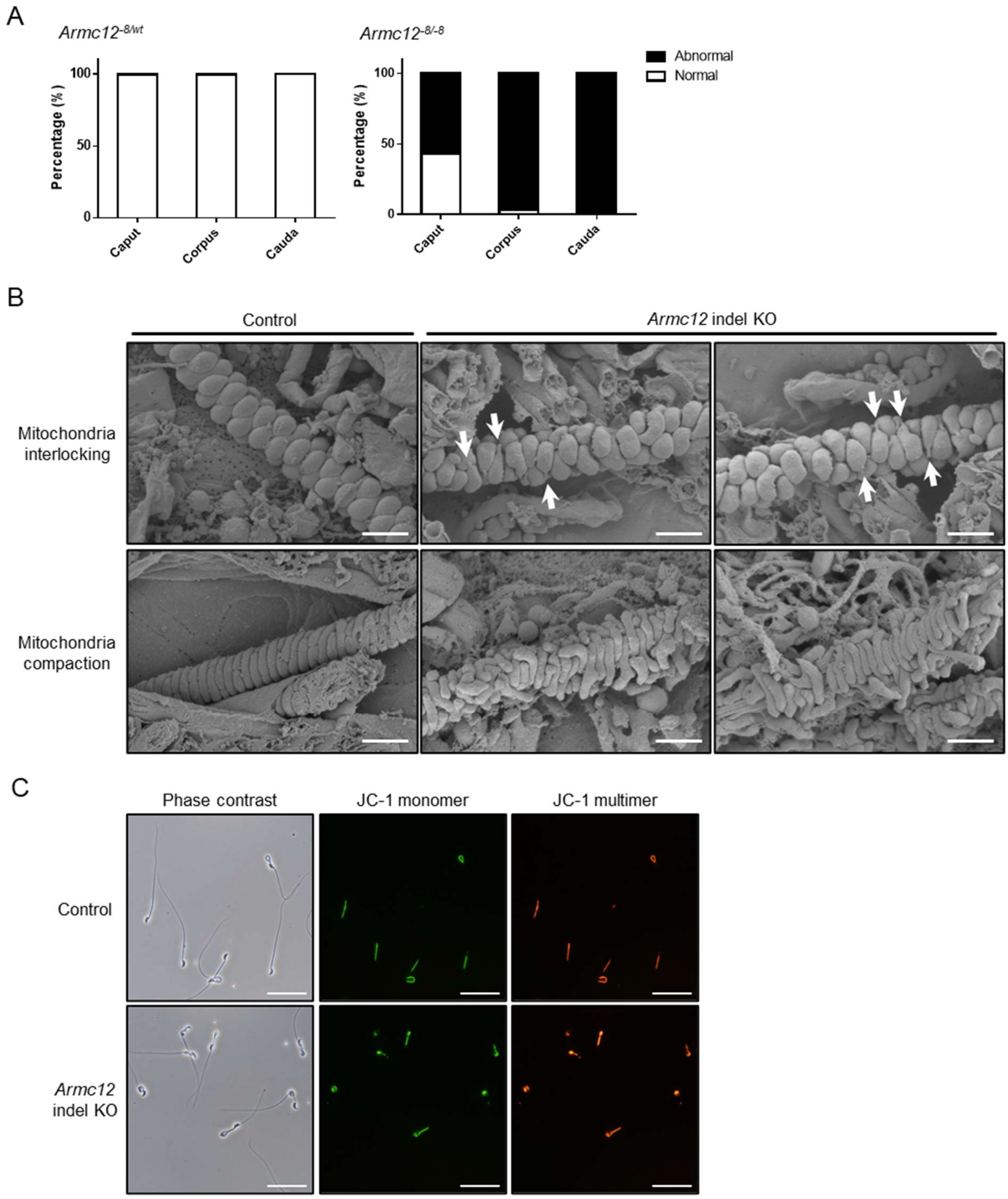

Fig. S4. Morphological abnormalities in *Armc12*-null spermatids. (*A*) Graphs indicate frequencies of abnormal spermatozoa collected from the three sections of epididymis (caput, corpus and cauda). (*B*) Various *Armc12*-null spermatids during spermiogenesis analyzed by SEM. Arrows indicate exposed mitochondrial tips. Scale bars are 1.0 μm. (*C*) Mitochondrial activities assessed by fluorescence of JC-1. When the membrane potential of inner mitochondrial membrane is high, JC-1 emits orange (590 nm) light (JC-1 multimer), while green (530 nm) light is emitted at low membrane potential (JC-1 monomer). Scale bars are 50 μm.

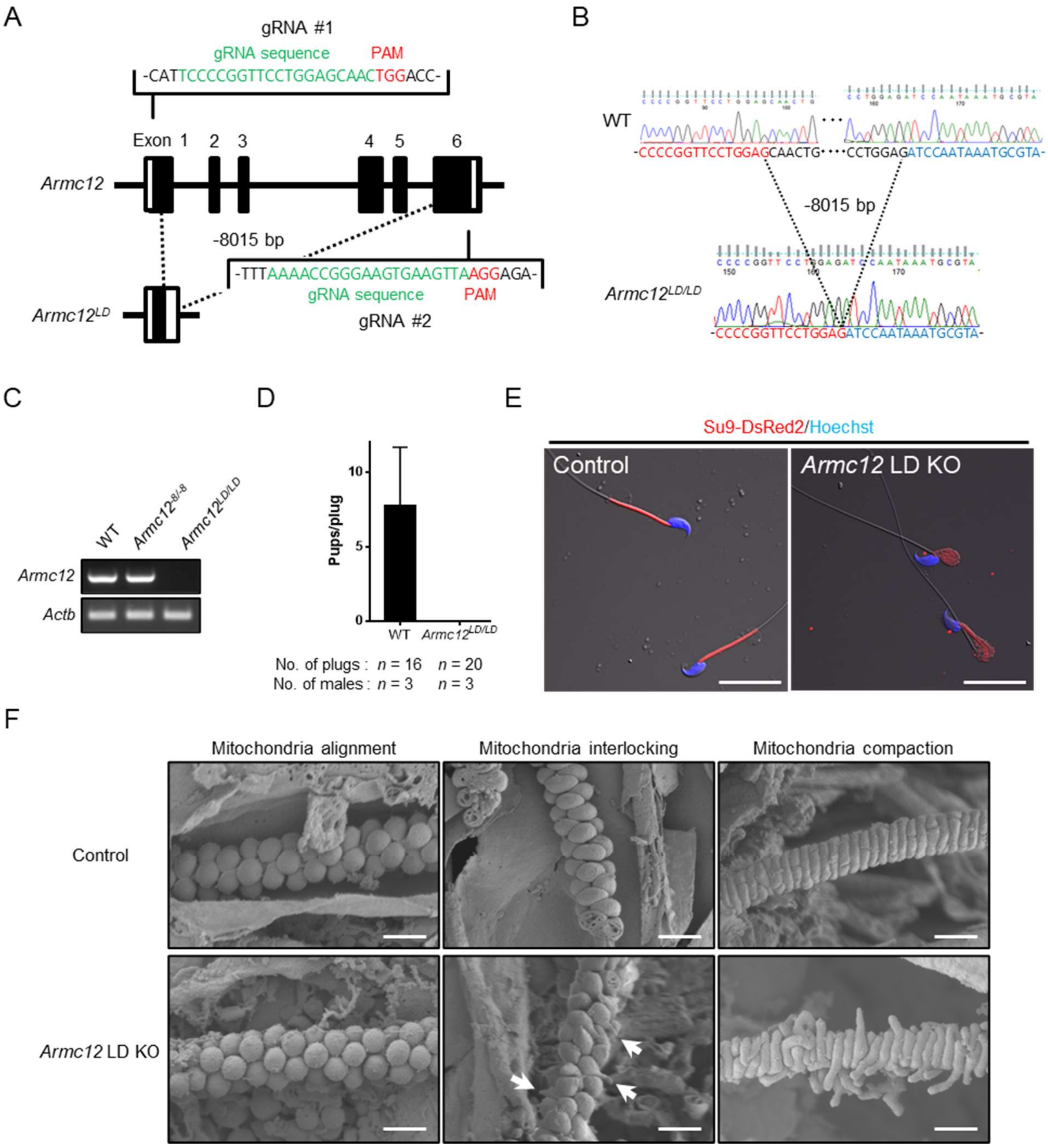

Fig. S5. *Armc12* large deletion mice have absence of an *Armc12* transcript and phenocopy *Armc12* indel KO mice. (*A*) Design of guide RNAs for generating *Armc12* LD KO mice. Guide sequences are highlighted in green and PAM motif are highlighted in red, respectively. (*B*) Control and *Armc12*$^{LD/LD}$ alleles. Black letters indicate deleted site. (*C*) RT-PCR amplification of the full-length *Armc12* transcript was performed using WT, *Armc12* indel KO and *Armc12* LD KO testis RNA. *Actb* was used as a loading control. (*D*) Pregnancy rate of control and *Armc12* LD KO male mice. (*E*) Sperm morphology of control and *Armc12* LD KO mice with RBGS transgenes, which express mitochondria-targeted DsRed2 (red). Nuclei were stained with Hoechst 33342 (blue). Scale bars are 20 μm. (*F*) Mitochondrial sheath development during spermiogenesis observed by SEM. Arrows indicate exposed mitochondrial tips. Scale bars are 1.0 μm.

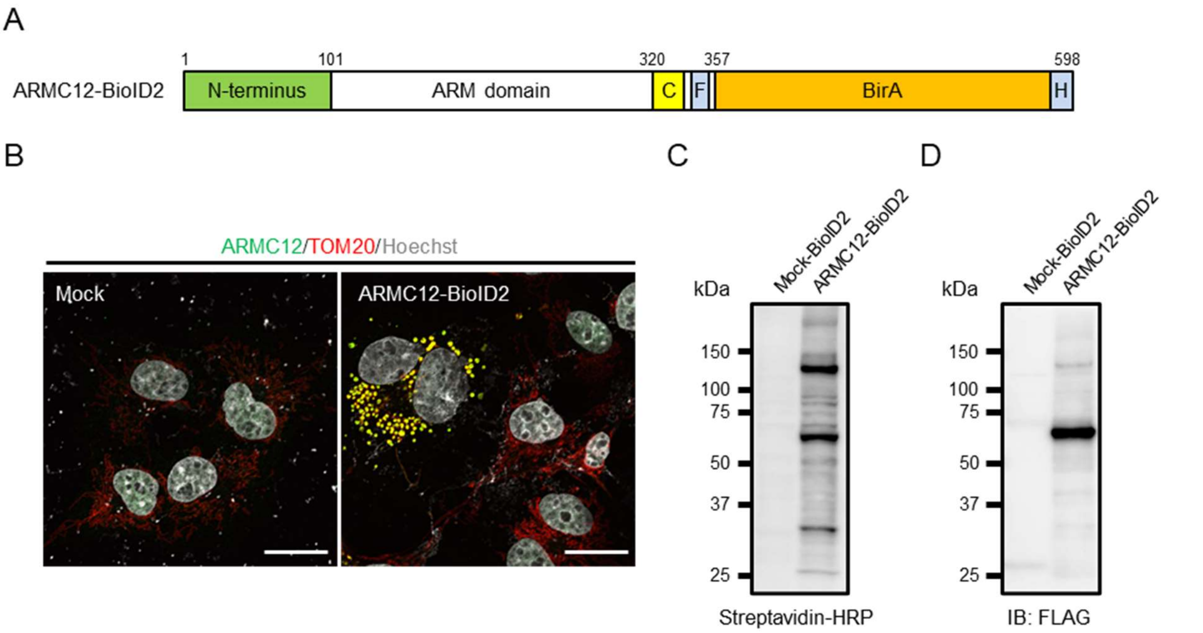

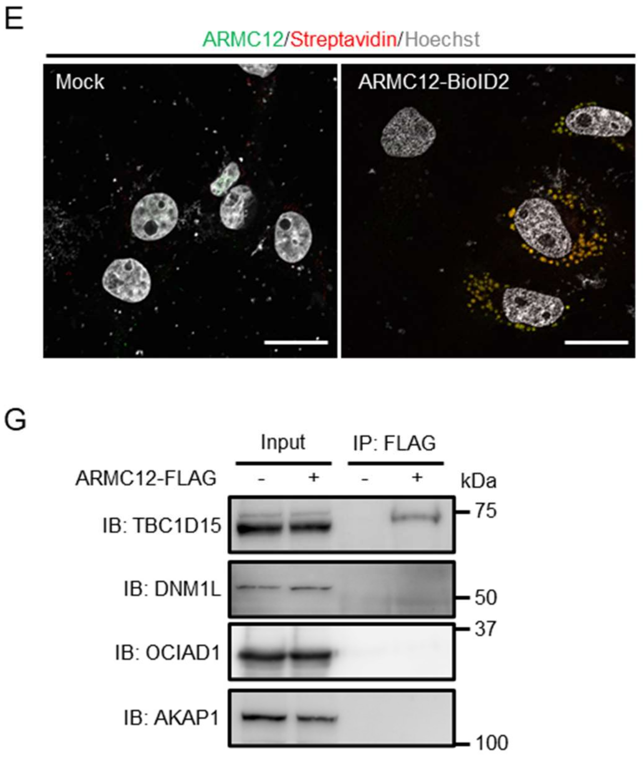

F

# Protein name	Quantitative Value		Ratio
	Armc12 -BioID2	Mock-BioID2	
1 TBC1D15	22.0	0	-
2 MAVS	16.7	0	-
3 HECTD1	13.2	0	-
4 DNM1L	12.3	0	-
5 MAP3K7	11.4	0	-
6 ATP1A3	10.6	0	-
7 HSPD1	10.6	0	-
8 SARS	10.6	0	-
9 PRKDC	9.7	0	-
10 ERC1	8.8	0	-
11 RBSN	7.0	0	-
12 CLINT1	6.2	0	-
13 TP53BP2	6.2	0	-
14 TDRKH	6.2	0	-
15 XPO5	6.2	0	-
16 CIAPIN1	6.2	0	-
17 PALLD	5.3	0	-
18 SASS6	5.3	0	-
19 HK1	5.3	0	-
20 HK1	5.3	0	-
21 OCIAD1	5.3	0	-
22 SNAP29	5.3	0	-
23 ARFGAP2	5.3	0	-
24 AIFM1	5.3	0	-
25 PHKA1	5.3	0	-
26 LDHB	5.3	0	-
27 SMG9	5.3	0	-
28 ARMC12-BioID2	141.8	4.6	30.6
29 AKAP1	57.2	2.3	24.7
30 FANCI	12.3	1.2	10.7

Fig. S6. ARMC12 interacts with TBC1D15 to regulate mitochondrial dynamics in cultured cells. (*A*) Schematic representation of BirA-fused ARMC12 vector (ARMC12-BioID2). HA-tagged BirA

was linked posterior to the FLAG-tagged ARMC12. Green and yellow boxes show the N-terminus and C-terminus of ARMC12, respectively. Light blue boxes indicate either the FLAG tag (F) or HA tag (H). A four-aa spacer is inserted between the FLAG tag and BirA. (*B*) COS-7 cells transiently expressing BirA-fused ARMC12 protein were stained with FLAG to visualize ARMC12 (green), and TOM20 (red) to visualize mitochondria. Hoechst 33342 (white) was used to visualize the nuclei. Scale bar are 20 µm. (*C* and *D*) HEK293T cells were transfected with BirA-fused ARMC12 plasmids. Biotinylated proteins were blotted using an HRP-conjugated streptavidin (*C*), and BirA-fused ARMC12 was immunoblotted using anti-FLAG antibody (*D*). (*E*) COS-7 cells transiently expressing BirA-fused ARMC12 protein were stained with FLAG to visualize ARMC12 (green), and labeled with Alexa Fluor 546-conjugated Streptavidin (red). Hoechst 33342 (white) was used to visualize the nuclei. Scale bar are 20 µm. (*F*) List of identified biotinylated-proteins by MS analysis *in vitro*. HEK293T cells were transfected with BirA-fused ARMC12 plasmids. Biotinylated proteins were pulled down using streptavidin beads and identified using MS analysis. Proteins either identified only in ARMC12-BioID2 (quantitative value >5.0) or highly enriched in ARMC12-BioID2 (ratio >10 as compared with mock-BioID2 transfection) are listed. (*G*) Co-IP followed by WB analysis were performed using lysates collected from HEK293T cells transfected with FLAG-tagged ARMC12. Immunoprecipitated proteins by anti-FLAG antibody were analyzed by WB using anti-TBC1D15, DNM1L, OCIAD1 and AKAP1 antibodies.

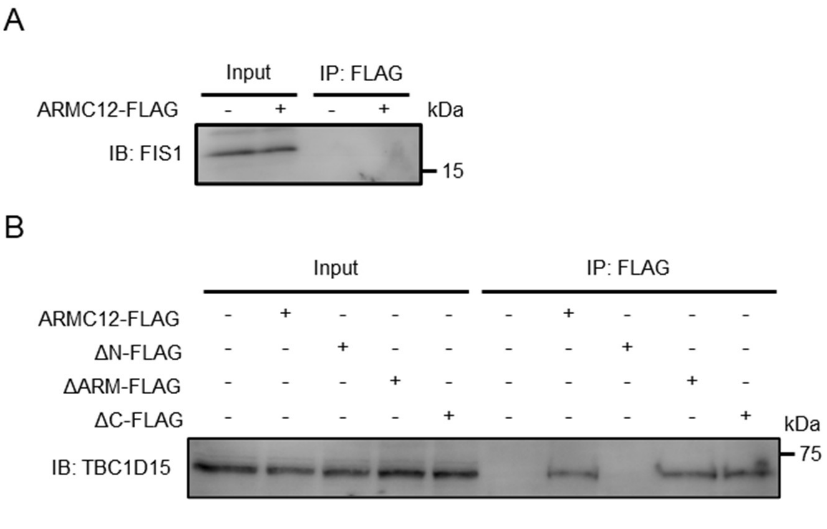

Fig. S7. ARMC12 interacts with the N-terminus of TBC1D15 to regulate mitochondrial dynamics in cultured cells. (*A*) HEK293T cells were transfected with FLAG-tagged ARMC12. Cell lysates were subjected to immunoprecipitation using an anti-FLAG antibody, and then analyzed by immunoblotting using anti-FIS1 antibody. (*B*) The interaction between various truncated ARMC12 and TBC1D15 were confirmed by co-IP followed by WB analysis using anti-FLAG, and TBC1D15 antibodies.

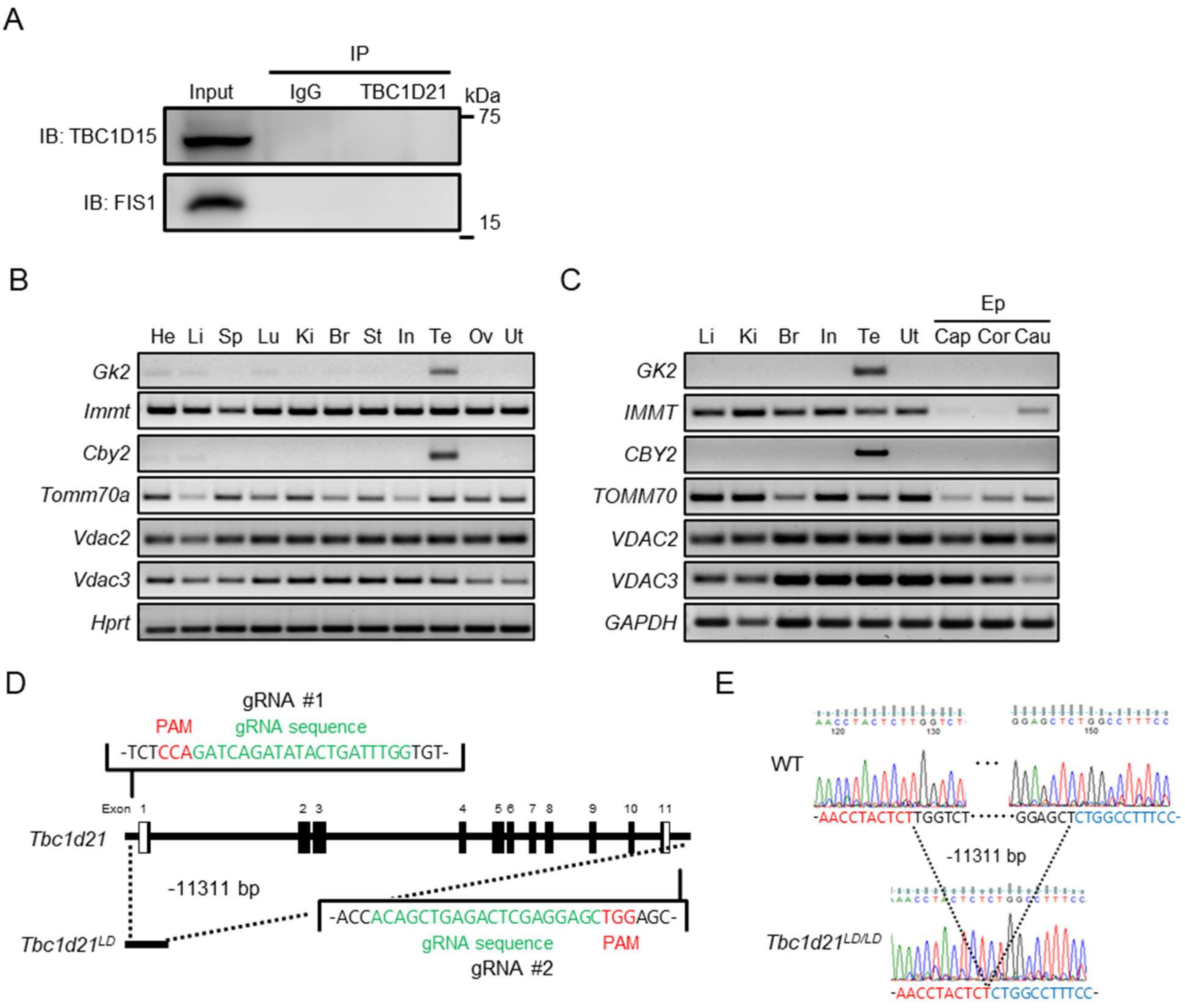

Fig. S8. There is no evidence for an ARMC12 and FIS1 interaction in testicular germ cells. (*A*) Co-IP followed by western blot analysis were performed using lysates collected from WT mouse TGC. Immunoprecipitated proteins by anti-TBC1D21 antibody were analyzed by WB using anti-TBC1D15 and FIS1 antibodies. IgG was used as a negative control for the IP. (*B*) RT-PCR for *Gk2, Immt, Cby2, Tomm70a, Vdac2* and *Vdac3* from various mouse tissues (He, heart; Li, liver; Sp, spleen; Lu, lung; Ki, kidney; Br, brain; St, stomach; In, intestine; Te, testis; Ov, ovary; Ut, uterus). *Hprt* as a control. (*C*) RT-PCR for *GK2, IMMT, CBY2, TOMM70, VDAC2* and *VDAC3* from various human tissues (Li, liver; Ki, kidney; Br, brain; In, intestine; Te, testis; Ut, uterus; Ep, epididymis; Cap, caput; Cor, corpus; Cau, Cauda). *GAPDH* as a control. (*D*) Design of guide RNAs for generating *Tbc1d21* LD KO mice. Guide sequences are highlighted in green and PAM motif are highlighted in red, respectively. (*E*) Control and *Tbc1d21*$^{LD/LD}$ alleles. Black letters indicate deleted site.

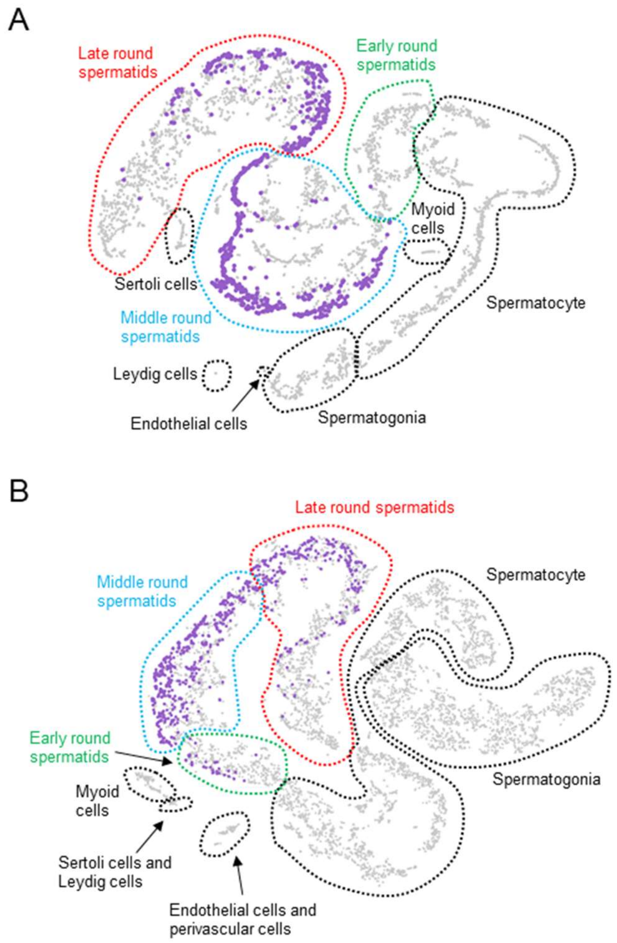

Fig. S9. Single cell RNA sequence analysis revealed that *Armc12* (*ARMC12*) transcripts express in spermatids both mouse and human. (*A*) Testicular *Armc12*-expressing cell population (purple dot) in mouse identified using single cell RNA sequence data. (*B*) Testicular *ARMC12*-expressing cell population (purple dot) in human identified using single cell RNA sequence data.

Table S1. The ID of mouse line used in this study.

Common name	Genotype	Strain name	RBRC No.	CARD ID
Armc12 indel KO	-8	B6D2-*Armc12*^{em1Osb}	09789	2436
Armc12-FLAG KI	FLAG	STOCK *Armc12*$^{em2(Armc12/FLAG)Osb}$	10338	2708
Armc12 LD KO	-8,015	B6D2-*Armc12*^{em3Osb}	10813	2793
Tbc1d21 KO	-11,311	STOCK *Tbc1d21*^{em1Osb}	11015	2922
RBGS	Tg	B6D2-Tg(CAG/Su9-DsRed2,Acr3-EGFP)RBGS002Osb	03743	1268

Table S2. Primer list.

Purpose	Primer sequence	Annealing temperature (°C)	Cycles	Sequencing primer
mouse *Armc12* RT-PCR	Fw: TGCATCAAGGTACTGGAACTG<br>Rv: GCTGTGAAGACTGGAAGAGG	50	30	-
mouse *Hprt* RT-PCR	Fw: TGGATATGCCCTTGACTATAATGAG<br>Rv: TGGCAACATCAACAGGACTC	50	35	-
mouse *Tbc1d15* RT-PCR	Fw: AGCAATTTCCCTACAGATGGC<br>Rv: TGTCATCTTTGGCTTCACTGG	58	35	-
mouse *Tbc1d21* RT-PCR	Fw: TCGATGAGAATGGTCACTTGG<br>Rv: CTCATACATCTGGCACAAGGAG	58	35	-
mouse *Fis1* RT-PCR	Fw: GGCTCTAAAGTATGTGCGAGG<br>Rv: AGGATTTGGACTTGGAGACAG	58	35	-
mouse *Gk2* RT-PCR	Fw: AGTCCACTGACCACTTTCATG<br>Rv: CTTGCTCCACCCATCCTTC	58	30	-
mouse *Immt* RT-PCR	Fw: AAGCGGACCTTTGACTCTG<br>Rv: ACTTCAGTTCCTGTTCCTGC	58	35	-
mouse *Cby2* RT-PCR	Fw: CGGATGGAGATGCTTATCGAAG<br>Rv: CTGGTCTTGGTGTCTATGTCTG	58	30	-
mouse *Tomm70a* RT-PCR	Fw: ACTCAGGACTTCAACATGGC<br>Rv: GCAAAACACTTCTGAGCCTG	58	35	-
mouse *Vdac2* RT-PCR	Fw: TGGAACACCGATAACACTCTG<br>Rv: AGTCAACATCACAGCCGAG	58	35	-
mouse *Vdac3* RT-PCR	Fw: ATAAGGTCTGCAACTATGGGC<br>Rv: TGCCGAGACTAAAACAATCCC	58	35	-
mouse full-length *ARMC12* RT-PCR	Fw: AGCCACAAGGCAGCACGG<br>Rv: TTGCTTCCGGAGGCCTCG	65	35	-
human *ARMC12* RT-PCR	Fw: AACAGGATGAGTATGCCAAG<br>Rv: GAAGGTTGTTGAGGAGTCTGAG	50	35	-
human *GAPDH* RT-PCR	Fw: AATCCCATCACCATCTTCCAG<br>Rv: ATGACCCTTTTGGCTCCC	50	30	-
human *TBC1D15* RT-PCR	Fw: TGAAGGCCAAGATAATCCAGG<br>Rv: CTGATGCATTTGGTCCATGTAAG	58	35	-
human *TBC1D21* RT-PCR	Fw: CCTGCTCCTGAGTTACGTCTG<br>Rv: AGCATGTCTAGGTTCTTGGC	58	35	-
human *FIS1* RT-PCR	Fw: TGACATCCGTAAAGGCATCG<br>Rv: AGTTCCTTGGCCTGGTTG	58	35	-
human *GK2* RT-PCR	Fw: TGAAATTCGTTATGCCACATGG<br>Rv: TGGGACTCCATAGCTGGTAT	58	30	-
human *IMMT* RT-PCR	Fw: TGTCTCTGTGGGAAGTTTGTC<br>Rv: TGGTTTTCTCTACACTTTCCCG	58	35	-
human *CBY2* RT-PCR	Fw: CATGGAAATGCTCATCGAGG<br>Rv: TTCAATAGGTACGAACTGGCTG	58	35	-
human *TOMM70* RT-PCR	Fw: ACCTTCTACCTGCTTATTGGC<br>Rv: TGCATTCTGAGGATCGATGTC	58	35	-
human *VDAC2* RT-PCR	Fw: GGAACACTGATAACACTCTGGG<br>Rv: TCAAAGTCAACATCACAACCAAG	58	35	-
human *VDAC3* RT-PCR	Fw: CTATGGACTTACCTTCACCCAG<br>Rv: GCCAACACTAAAACAATCCCG	58	35	-
Armc12 Indel genotyping	Fw: GCTGATTCTAGTACCCTGGTTCTGG<br>Rv: GTACAGCAGGTATAAGGCCC	65	40	GCTGATTCTAGTACCCTGGTTCTGG
Armc12 large deletion genotyping	Fw: TGTTCTCTCCAGGCCTAGCT<br>Rv: GAACGACTTGGCTGGTGAGA	65	40	TGATGAACAGCACCTCCACC
Armc12 WT allele genotyping	Fw: GACATCGACTTGGTGGCTGA<br>Rv: CGGATCTTACCCAGGCTCAC	65	40	-
Armc12-FLAG genotyping	Fw: CATCATCTCTCCGGCCTCAC<br>Rv: CACACACACATCTCCCCCTC	65	40	TGATGAACAGCACCTCCACC
Tbc1d21 WT allele genotyping	Fw: AGTTTGCCCAAGACCTTGCT<br>Rv: TGGAACACACACAGAAGCCA	60	40	-
Tbc1d21 large deletion genotyping	Fw: AGTTTGCCCAAGACCTTGCT<br>Rv: CAGGCTGGGCATGGAACTTA	60	40	AGTTTGCCCAAGACCTTGCT
Screening of FLAG-tagged *Armc12* allele	Fw: CATCATCTCTCCGGCCTCAC<br>Rv: TCATCCTTGTAGTCGCTGCC	65	40	-
Gene cloning of mouse *Armc12*	Fw: CTGCAGGCCGCCATGGGCAAGACCATTCCCCGG<br>Rv: GATATCGCTGCCGCCCTTCCCGGTTTTAAAGCAGGAATG	65	40	-
Inverse PCR for ΔN-FLAG	Fw: CATGGCGGCCTGCAGGGATC<br>Rv: GAGGCTGAGGCCTCTTCTTG	68	7	-
Inverse PCR for ΔARM-FLAG	Fw: CAGCAAGTACACGCAGCGGG<br>Rv: CAGGACCTGGGATCCCGACC	68	7	-
Inverse PCR for ΔC-FLAG	Fw: GGGACACTGCAAGCTCACTATG<br>Rv: GGCGGCAGCGATATCGACTA	68	7	-

Table S3. Antibody list.

Immunoblot analysis

Antibody	Clone No.	Host species	Catalog No.	Company	Dilution
anti-ACTB	AC-15	Mouse	ab6276	abcam	1:5000
anti-ADAM3	7C1.2	Mouse	MAB19291	Merck	1:2000
anti-AKAP1	polyclonal	Rabbit	A301-379A	Bethyl	1:500
anti-COX IV	EPR9442(ABC)	Rabbit	ab202554	abcam	1:2000
anti-DNM1L (DRP1)	polyclonal	Rabbit	12957-1-AP	Proteintech	1:1000
anti-FIS1	polyclonal	Rabbit	10956-1-AP	Proteintech	1:500
anti-FLAG	polyclonal	Rabbit	PM020	MBL	1:1000
anti-GAPDH	14C10	Rabbit	2118	Cell Signaling	1:1000
anti-GK2	polyclonal	Rabbit	ab96818	abcam	1:500
anti-MIC60 (IMMT, Mitofilin)	polyclonal	Rabbit	10179-1-AP	Proteintech	1:1000
anti-OCIAD1	1C10C3	Mouse	66698-a-Ig	Proteintech	1:2000
anti-SPERT (CBY2)	polyclonal	Rabbit	17826-1-AP	Proteintech	1:1000
anti-TBC1D15	polyclonal	Rabbit	17252-1-AP	Proteintech	1:500
anti-TBC1D21	polyclonal	Rabbit	-	In house	1:1000
anti-VDAC2	polyclonal	Rabbit	11663-1-AP	Proteintech	1:500
anti-VDAC3	polyclonal	Rabbit	14451-1-AP	Proteintech	1:500
anti-TOM70	polyclonal	Rabbit	14528-1-AP	Proteintech	1:1000
anti-ZPBP	G176	Goat	-	In house	1:2000

Immunofluorescence analysis

Antibody	Clone No.	Host species	Catalog No.	Company	Dilution
anti-FLAG	polyclonal	Rabbit	F7425	Merck	1:1000
anti-FLAG	polyclonal	Rabbit	PM020	MBL	1:200
anti-IZUMO1	KS64-125	Rat	-	In house	1:1000
anti-TOM20	F-10	Mouse	sc-17764	Santa Cruz	1:500

Immunoelectron microscopy

Antibody	Clone No.	Host species	Catalog No.	Company	Dilution
anti-FLAG	M2	Mouse	F1804	Sigma	1:150

Immunoprecipitation

Antibody	Clone No.	Host species	Catalog No.	Company
anti-FIS1	polyclonal	Rabbit	10956-1-AP	Proteintech
anti-FLAG	FLA-1	Mouse	M185-3L	Sigma-Aldrich
anti-TBC1D21	polyclonal	Rabbit	-	In house

Movie S1 (separate file). Spermatozoa collected from *Armc12*$^{-8/wt}$ KO mice.

Movie S2 (separate file). Spermatozoa collected from *Armc12*$^{-8/-8}$ KO mice.

Movie S3 (separate file). Spermatozoa collected from *Armc12*$^{LD/wt}$ KO mice.

Movie S4 (separate file). Spermatozoa collected from *Armc12*$^{LD/LD}$ KO mice.

Movie S5 (separate file). Mitochondrial aggregation observed in COS-7 cells after FLAG-tagged *Armc12* transfection.

Arrow indicates a cell that shows mitochondrial aggregation.

SI References

1. Hasuwa H, *et al.* (2010) Transgenic mouse sperm that have green acrosome and red mitochondria allow visualization of sperm and their acrosome reaction in vivo. *Exp. Anim.* 59(1):105-107.
2. Langmead B, Trapnell C, Pop M, & Salzberg SL (2009) Ultrafast and memory-efficient alignment of short DNA sequences to the human genome. *Genome Biol.* 10(3):R25.
3. Mashiko D, *et al.* (2013) Generation of mutant mice by pronuclear injection of circular plasmid expressing Cas9 and single guided RNA. *Sci. Rep.* 3:3355.
4. Ho Y, Wigglesworth K, Eppig JJ, & Schultz RM (1995) Preimplantation development of mouse embryos in KSOM: Augmentation by amino acids and analysis of gene expression. *Mol. Reprod. Dev.* 41(2):232-238.
5. Abbasi F, *et al.* (2018) RSPH6A is required for sperm flagellum formation and male fertility in mice. *J. Cell Sci.* 131(19).
6. Oji A, *et al.* (2016) CRISPR/Cas9 mediated genome editing in ES cells and its application for chimeric analysis in mice. *Sci. Rep.* 6:31666.
7. Fujihara Y, Kaseda K, Inoue N, Ikawa M, & Okabe M (2013) Production of mouse pups from germline transmission-failed knockout chimeras. *Transgenic Res.* 22(1):195-200.
8. Larkin MA, *et al.* (2007) Clustal W and Clustal X version 2.0. *Bioinformatics* 23(21):2947-2948.
9. Toyoda Y & Yokoyama M (2016) The Early History of the TYH Medium for in vitro Fertilization of Mouse Ova. *Journal of Mammalian Ova Research* 33(1):3-10.
10. Shimada K, Kato H, Miyata H, & Ikawa M (2019) Glycerol kinase 2 is essential for proper arrangement of crescent-like mitochondria to form the mitochondrial sheath during mouse spermatogenesis. *J Reprod Dev.* 65(2):155-162.
11. Tokuhiro K, Ikawa M, Benham AM, & Okabe M (2012) Protein disulfide isomerase homolog PDILT is required for quality control of sperm membrane protein ADAM3 and male fertility. *Proc. Natl. Acad. Sci. U. S. A.* 109(10):3850-3855.
12. Mukai C & Okuno M (2004) Glycolysis Plays a Major Role for Adenosine Triphosphate Supplementation in Mouse Sperm Flagellar Movement. *Biol. Reprod.* 71(2):540-547.
13. Lawitts JA & Biggers JD (1993) [9] Culture of preimplantation embryos. *Methods Enzymol.*, (Academic Press), Vol 225, pp 153-164.
14. Yamaguchi R, Yamagata K, Ikawa M, Moss SB, & Okabe M (2006) Aberrant distribution of ADAM3 in sperm from both angiotensin-converting enzyme (Ace)- and calmegin (Clgn)-deficient mice. *Biol. Reprod.* 75(5):760-766.
15. Fujihara Y, *et al.* (2012) SPACA1-deficient male mice are infertile with abnormally shaped sperm heads reminiscent of globozoospermia. *Development* 139(19):3583-3589.
16. Lin Y-N, Roy A, Yan W, Burns KH, & Matzuk MM (2007) Loss of Zona Pellucida Binding Proteins in the Acrosomal Matrix Disrupts Acrosome Biogenesis and Sperm Morphogenesis. *Mol. Cell. Biol.* 27(19):6794-6805.
17. Blunsom NJ, Gomez-Espinosa E, Ashlin TG, & Cockcroft S (2018) Mitochondrial CDP-diacylglycerol synthase activity is due to the peripheral protein, TAMM41 and not due to the integral membrane protein, CDP-diacylglycerol synthase 1. *Biochimica et Biophysica Acta (BBA) - Molecular and Cell Biology of Lipids* 1863(3):284-298.
18. Isotani A, *et al.* (2005) Genomic imprinting of XX spermatogonia and XX oocytes recovered from XX<-->XY chimeric testes. *Proc. Natl. Acad. Sci. U. S. A.* 102(11):4039-4044.
19. Russell L, Ettlin R, Sinha Hikim A, & Clegg E (1990) Histological and histopathological evaluation of the testis. 1990. (Cache River Press).
20. Noda T, *et al.* (2020) Sperm proteins SOF1, TMEM95, and SPACA6 are required for sperm−oocyte fusion in mice. *Proceedings of the National Academy of Sciences* 117(21):11493-11502.
21. Niwa H, Yamamura K, & Miyazaki J (1991) Efficient selection for high-expression transfectants with a novel eukaryotic vector. *Gene* 108(2):193-199.
22. Le Sage V, Cinti A, & Mouland AJ (2016) Proximity-Dependent Biotinylation for Identification of Interacting Proteins. *Curr. Protoc. Cell Biol.* 73(1):17.19.11-17.19.12.

23. Meier F, *et al.* (2015) Parallel Accumulation–Serial Fragmentation (PASEF): Multiplying Sequencing Speed and Sensitivity by Synchronized Scans in a Trapped Ion Mobility Device. *J. Proteome Res.* 14(12):5378-5387.
24. Searle BC (2010) Scaffold: A bioinformatic tool for validating MS/MS-based proteomic studies. *PROTEOMICS* 10(6):1265-1269.
